# Genotype distribution-based inference of collective effects in genome-wide association studies: insights to age-related macular degeneration disease mechanism

**DOI:** 10.1186/s12864-016-2871-3

**Published:** 2016-08-30

**Authors:** Hyung Jun Woo, Chenggang Yu, Kamal Kumar, Bert Gold, Jaques Reifman

**Affiliations:** 1 Biotechnology High Performance Computing Software Applications Institute, Telemedicine and Advanced Technology Research Center, U.S. Army Medical Research and Materiel Command, Fort Detrick, Maryland USA; 2 Laboratory of Genomic Diversity, National Cancer Institute, Frederick, Maryland USA

**Keywords:** Genome-wide association, Machine learning, Epistasis, Single-nucleotide polymorphism, Age-related macular degeneration

## Abstract

**Background:**

Genome-wide association studies provide important insights to the genetic component of disease risks. However, an existing challenge is how to incorporate collective effects of interactions beyond the level of independent single nucleotide polymorphism (SNP) tests. While methods considering each SNP pair separately have provided insights, a large portion of expected heritability may reside in higher-order interaction effects.

**Results:**

We describe an inference approach (discrete discriminant analysis; DDA) designed to probe collective interactions while treating both genotypes and phenotypes as random variables. The genotype distributions in case and control groups are modeled separately based on empirical allele frequency and covariance data, whose differences yield disease risk parameters. We compared pairwise tests and collective inference methods, the latter based both on DDA and logistic regression. Analyses using simulated data demonstrated that significantly higher sensitivity and specificity can be achieved with collective inference in comparison to pairwise tests, and with DDA in comparison to logistic regression. Using age-related macular degeneration (AMD) data, we demonstrated two possible applications of DDA. In the first application, a genome-wide SNP set is reduced into a small number (∼100) of variants via filtering and SNP pairs with significant interactions are identified. We found that interactions between SNPs with highest AMD association were epigenetically active in the liver, adipocytes, and mesenchymal stem cells. In the other application, multiple groups of SNPs were formed from the genome-wide data and their relative strengths of association were compared using cross-validation. This analysis allowed us to discover novel collections of loci for which interactions between SNPs play significant roles in their disease association. In particular, we considered pathway-based groups of SNPs containing up to ∼10, 000 variants in each group. In addition to pathways related to complement activation, our collective inference pointed to pathway groups involved in phospholipid synthesis, oxidative stress, and apoptosis, consistent with the AMD pathogenesis mechanism where the dysfunction of retinal pigment epithelium cells plays central roles.

**Conclusions:**

The simultaneous inference of collective interaction effects within a set of SNPs has the potential to reveal novel aspects of disease association.

**Electronic supplementary material:**

The online version of this article (doi:10.1186/s12864-016-2871-3) contains supplementary material, which is available to authorized users.

## Background

A key focus of modern genetic research is the relationship between genomic variations and phenotypes, including susceptibilities to common diseases [1]. Recent advances in genome-wide association studies (GWAS) have greatly enhanced our understanding of such genotype-phenotype relationships [2–9]. In many cases, however, a large portion of the expected heritability information remains to be discovered. It has recently been shown that meta-analyses involving increasingly large sample sizes can yield many additional loci of statistical significance [10, 11]. Another potential source of such ‘missing heritability’ is the contribution of rare variants not detected by population-based genotyping platforms. Recent studies based on exome and whole-genome sequencing data combined with statistical tests including burden tests [12], C-alpha test [13], and sequence kernel association test [14] are beginning to address such possibilities. It is also expected, however, that the limitation of independent single nucleotide polymorphism (SNP) analyses, where each locus is considered separately to evaluate its association with disease using trend tests or logistic regression models [15], and possible effects of epistasis also contribute to the limited degree of biological effects uncovered so far.

Many studies have addressed the issue of incorporating such inter-variant interactions, or epistasis, in GWAS [16, 17]. Main approaches include machine-learning techniques [18–23], entropy-based methods [24], principal component analyses [25–27], and the genome-wide interaction analysis considering all distinct pairs of SNPs [28–31]. One useful strategy, in particular, is to extend parametric models to many SNPs that have been suitably selected, and inferring interaction effects under a multivariate statistical setting. Previous works within this framework include those based on lasso-penalized logistic regression [32, 33]. Under the setting of inference on many interacting SNPs, the dimensionality of the underlying model is of the order of *m*^2^, where *m* is the number of SNPs that are considered simultaneously, with *m*=1 and 2 corresponding to the independent-SNP and pairwise tests, respectively. To prevent model overfitting, high-dimensional inference with limited sample sizes requires regularization, whose values can be determined by cross-validation. Ayers and Cordell performed a comprehensive study of the performance of different penalizer choices on noninteracting SNP inferences [34].

This class of methods within the context of GWAS so far exclusively used logistic (or linear) regression analyses for case-control (or quantitative phenotype) data, which parallels their similarly widespread adoption in the general statistical learning literature. One may note, however, that the actual training data sets in GWAS are collected from case and control populations with distinct genotype distributions. The likelihood of the data to be maximized for inference is given by the joint probability of both genotypes (predictor variables) and phenotypes (response variables). In (logistic) regression, this joint probability is replaced by the probability of phenotypes conditional to genotypes, and the marginal probability of genotypes is assumed to be uniform.

In statistical learning, discriminant analysis is another widely used option for classifying continuous random variables in addition to logistic regression [35–37]. This class of inference methods offers alternative approaches that fully model the joint distribution of predictor and response variables (Section 4.4.5 in Hastie et al. [37]) at the expense of assuming specific predictor distributions (usually multivariate normal distributions). It has been estimated that, for continuous variables, the accuracy of logistic regression models can be lower by ∼30 *%* than that of discriminant analyses for a given sample size [35, 37].

Genotype distributions within populations from which GWAS samples are collected are also far from uniform, and it is of interest to examine the utility of discriminant analysis-type approaches to disease association inference under high-dimensional settings, which is the main focus of this paper. The standard discriminant analysis, however, is applicable only for continuous variable predictors. A related approach, the discriminant analysis of principal components by Jombart et al. [38], applies discriminant analysis to principal components (continuous variables) of allele frequencies for unsupervised learning of population structures. We report here, as a major innovation, an adaptation of discriminant analysis to the case of discrete genotype data (*discrete* discriminant analysis; DDA).

Our inference includes the causal effects of both marginal single-SNP terms and their interactions. These effects are estimated simultaneously, rather than separately as in independent-SNP and pairwise analyses. We refer to such combined effects of single-SNP and interaction contributions as the *collective* effects of disease association. This level of description is analogous to that of the logistic regression inference performed by Wu et al. [33] in terms of the nature of SNP effects included in the modeling. Association studies have two distinct but related goals: inference and prediction. In inference (also known as feature selection), one aims to identify a subset of SNPs that are deemed to be causal, while in prediction, the goal is to apply the trained model and predict the disease status of unknown samples. Independent-SNP analyses widely performed in GWAS, either based on trend tests or logistic regression models with marginal SNP effects only, are mainly geared toward inference. In contrast, the penalized logistic regression including collective effects [33] is more suited to prediction, because the disease risk parameters are optimized directly via maximum likelihood without reference to population structures.

Our method offers a comprehensive approach achieving both inference and prediction by training models to genotype distributions of case and control groups separately under penalizers. The regularization using cross-validation optimizes prediction capability, while for inference, we derived effective *p*-values of the overall single-SNP (we use this terminology to refer to the contribution each locus makes by itself to the overall association, usually in the presence of interactions) and interaction effects using likelihood ratio tests. To our knowledge, the performance comparison of interaction effect detection between pairwise tests and (logistic regression) collective inference has not been reported yet. Our results based on simulated data indicate that collective inference provides far higher sensitivity for interactions than pairwise tests. Compared to penalized logistic regression, DDA yielded further advantages in sensitivity and specificity.

Our current collective inference implementation allows for the maximum likelihood inference of systems containing up to ∼10^4^ SNPs. However, evaluating interaction *p*-values of SNP pairs by permutation resampling increases the computational cost by orders of magnitude, limiting the number of SNPs that can be considered in practice. In addition, the requirement for pre-selection of variants based, for example, on independent-SNP *p*-values, limits the possibility for discovering novel loci whose effects are significant only when interactions are taken into account. To deal more directly with genome-wide data in an unbiased fashion, we describe a second mode of DDA application where ∼10^6^ SNPs are grouped into (∼10^3^ or more) subsets based on phenotype-independent criteria (e.g., biological pathways), the collective inference is applied to each subset, and their relative importance in disease association is evaluated based on cross-validation prediction score. This protocol significantly expands the power of SNP-based pathway analysis beyond existing enrichment-based methodologies [39] by allowing for the incorporation of collective interaction effects within each pathway.

By applying our algorithm to the disease data of age-related macular degeneration (AMD) [40, 41], we demonstrate that the enhanced ability to account for interaction effects can translate into novel biological findings. AMD is a progressive degenerative disease affecting individuals in old age, characterized by the accumulation of deposits (drusen) in the retina or choroidal neovascularization, which can lead to vision loss. Genome-wide studies of AMD constitute one of the earliest and most successful applications of GWAS [2, 3, 40–43], where strong associations were detected and later validated at variants including those near complement pathway genes *CFH*, *C2*, and *C3*, in addition to the *ARMS2/HTRA1* loci. However, direct molecular mechanisms tying these associated loci into disease pathogenesis remain unclear. Using AMD case-control data, we first analyzed detailed interaction patterns within SNPs selected based on independent-SNP association strengths. These interactions were enriched in loci epigenetically active in tissues including adipocytes, mesenchymal stem cells, and the liver. We then applied DDA to pathway-based groups formed from genome-wide data and found high association with pathways involved in phospholipid synthesis, cellular stress response, apoptosis, and complement activation.

## Results and discussion

**Fig. 1:**
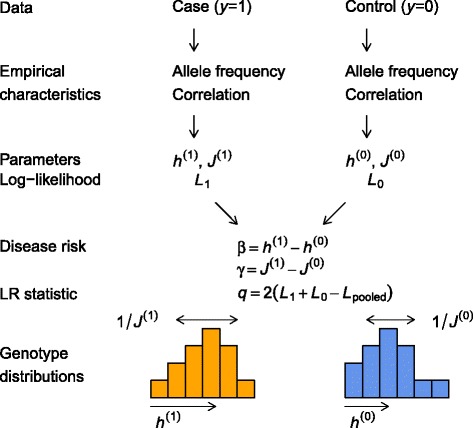
Discrete discriminant analysis algorithm. Empirical characteristics (allele frequency and correlation) of case (*y*=1) and control (*y*=0) data are used to fit their genotype distributions with parameters $h_{i}^{(y)}$ and $J_{ij}^{(y)}$, each roughly determining the position and width of the distribution. Disease risk parameters are given by their differences, whereas the likelihood ratio (LR) statistic *q* is obtained from the difference between the sum of two contributions and the corresponding pooled value

Our algorithm (DDA) extends the discriminant analysis to discrete genotype data. Its overall steps are summarized in Fig. 1 and described in Methods (see Additional file 1: Text S1 for more in-depth details).

### Independent SNPs

When interactions between the loci are turned off, DDA can be solved analytically (see Additional file 1: Text S1), whereas logistic regression is always numerical. We first compared this special case of DDA and logistic regression without interaction and found the odds ratio and power to be identical for all conditions for binary models (Additional file 2: Figure S1), which implies that the effect of marginal genotype distributions ignored in logistic regression is negligible for a single non-interacting locus. However, since DDA can yield *p*-values of each locus without numerical optimization, it leads to considerable computational speed-up even when interactions are not included.

### Simulation

We compared pairwise tests, logistic regression, and DDA in similarly well-controlled but high-dimensional conditions in which collective effects can play important roles. In the following, unless otherwise specified, logistic regression refers to the collective inference including both marginal and interaction terms and a penalizer (see Methods). We used simulated data that faithfully reflected prescribed genotype distributions of given sample sizes. A genotype distribution for a binary model with *m* loci has *m* single-SNP and *m*(*m*−1)/2 interaction parameters. We specified these parameters randomly from normal distributions, generated genotype samples of size *n* based on these distributions, performed pairwise marginal inference, logistic regression, and DDA, and compared inferred parameters with the true values (Additional file 3: Figure S2 shows examples for the dominant and genotypic models). Our simulated data include linkage disequilibrium (LD): if one approximates the genotype distribution as a continuous-variable normal distribution, the correlation within a single group (case or control) would be proportional to the matrix inverse of interaction parameters specified, and the overall *r*^2^ would correspond to the sample size-weighted average over case and control groups.

For a given sample, we first determined the optimal penalizer (*λ*) value by cross-validation. With increasing *λ*, the mean square error and the area under the curve (AUC) of the receiver operating characteristics generally showed a minimum and maximum, respectively, at *λ*^∗^ (Additional file 4: Figure S3). The value of *λ*^∗^ decreased as the sample size *n* increased. This trend implies that for small *n*, an aggressive regularization is needed (large *λ*^∗^) to minimize overfitting, while for larger *n*, more interaction terms are inferred with sufficient significance, leading to smaller *λ*^∗^.

**Fig. 2:**
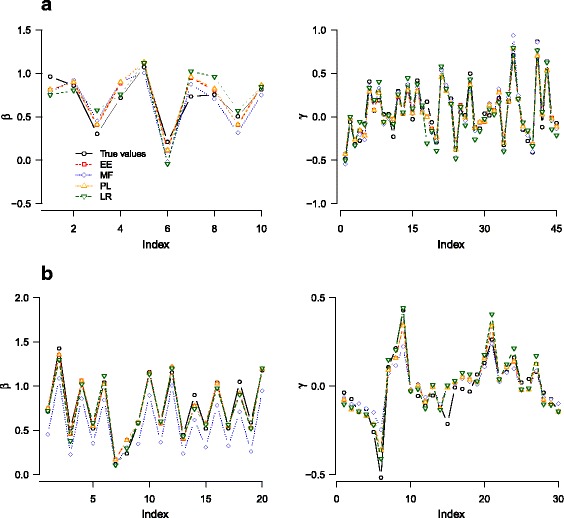
Inference accuracy, sensitivity, and specificity of pairwise and collective inference on simulated data. **a**–**b** The mean square error and AUC versus sample sizes using pairwise test (PW), logistic regression (LR), and the three methods of DDA (MF, PL, and EE). Simulated genotypes were generated for 10 SNPs with parameters ${\bar h}_{y}=(-1,-0.3)$, ${\bar J}=(0,0.1)$, *σ*_*h*_=*σ*_*J*_=0.2 (see Methods). **c**-**d** Analogous results for 20 SNPs with ${\bar h}_{y}=(-1,-1+\Delta h)$, ${\bar J}=(0,\Delta J)$, and *σ*_*h*_=*σ*_*J*_=0.2. We set *Δ* *h*=0.7, *Δ* *J*=0.5 for the first 4 SNPs and their interactions and *Δ* *h*=*Δ* *J*=0 otherwise. **e**-**f** Sensitivity and specificity of disease-associated interaction pairs. Simulated data were generated with parameters ${\bar h}=(-1,-1)$, ${\bar J}=(0.01,0.01)$, *σ*_*h*_=0.1, *σ*_*J*_=0.05 for *m*=10 SNPs, except the interaction between the first two SNPs, for which we set ${\bar J}=(0.01,0.11)$. Interaction *p*-values for all pairs were calculated either by PW or by regularization to determine *λ* ^∗^ followed by the construction of null distribution under *λ* ^∗^ (Additional file 5: Figure S4) for LR, PL, and EE. The distribution of *p*-values for the true causal interaction pair and those of non-causal pair (geometric mean) are shown in **e** and **f**, respectively. The dominant model was used in all cases

**Accuracy of inference** We compared results of pairwise marginal tests using PLINK [44], logistic regression, and three versions of DDA [exact enumeration; EE, pseudo-likelihood; PL, and mean field; MF (see Methods)] in two different simulation settings. In the first case (Fig. 2a–b), we used *m*=10 SNPs with parameters chosen such that all sites had relatively large and strong single-SNP and interaction effects. We used the dominant model in these simulations in order to facilitate sampling, which requires exhaustive enumeration of all genotypes (2^*m*^ and 3^*m*^ for binary and genotypic models, respectively). Pairwise tests derive odds ratios and *p*-values for each SNP pair separately, and the logarithm of the interaction odds ratio corresponds to the interaction parameter. The mean square error of pairwise inference decreased slightly from sample size *n*=10^2^ to 10^3^ but showed little improvement for larger sample sizes. Outcomes from logistic regression and DDA exhibited AUC values (maximized with respect to *λ* for each sample) increasing with *n* for *n*≤10^3^. The AUCs from logistic regression were slightly lower for *n*≤10^3^ than DDA and comparable in larger sample sizes. The mean square error of logistic regression and DDA steadily decreased (approximately linearly in log-log scale) over all *n* ranges examined. Error levels of DDA from three methods were similar to one another. When compared to logistic regression, the accuracy of DDA was comparable at larger *n* and better at smaller *n*. However, the logistic regression results showed much larger variances (with respect to different realizations of samples) for small *n* than DDA.

In the second setting (Fig. 2c–d), we enlarged the system to *m*=20 SNPs (EE omitted due to computational costs), and set the parameters such that only 4 SNPs contributed to disease association. The AUC values were smaller in comparison to the first setting for smaller *n*, which reflects a weaker overall strength of disease association, but otherwise showed similar trends. The accuracy of pairwise tests, logistic regression, and DDA also exhibited trends similar to simulations in Fig. 2a: both logistic regression and DDA significantly outperformed pairwise tests, while DDA consistently showed slightly better accuracy than logistic regression. The variances in mean square error were smaller than in the first setting, which suggests that these variances correlate with the number of causal SNPs. For *n*=10^2^, logistic regression results had a variance much larger than DDA for small *n*.

These simulations demonstrate that when both marginal single-SNP and interaction effects are included, the accuracy of collective inference approach is much higher than that of pairwise tests. The DDA generally provides a further edge for smaller sample sizes in comparison to logistic regression. The comparison of two different simulation settings in Fig. 2a–b and c–d demonstrates that this trend is not significantly altered with increases in the number of SNPs and the fraction of causal SNPs among them. The accuracy (inferred model parameters versus true values) remained at similar levels when the underlying model was changed from dominant to genotypic models (Additional file 3: Figure S2).

**Statistical tests** We then examined the performance of collective inference methods in evaluating the statistical significance of individual interactions. In GWAS, the significance of SNPs and their interactions are tested either by contingency table or likelihood ratio tests [15]. The presence of the penalizer *λ* complicates this approach in collective inference. In their study of lasso-penalized logistic regression collective inference, Wu et al. [45] adopted the approach of first selecting significant SNPs of a certain size using regularization, and then calculating *p*-values of interactions with the penalizer turned off. A disadvantage of this approach is that the information of the relative importance of each interactions reflected in the penalized model is lost when *λ* is set to zero.

The (analytic) likelihood ratio tests rely on the asymptotic distribution of the likelihood ratio statistic *q* (*q*_*i*_ and *q*_*ij*_ for a site *i* and pair *i*,*j*): as *n*→*∞*, the distribution of *q* under the null hypothesis approaches the *χ*^2^-distribution with degrees of freedom (d.f.) given by the change in the number of parameters between the null and alternative hypotheses [46]. In practice, however, with a finite *n*, the deviation from this asymptotic limit can be significant. We found the null distribution to show increasingly large deviations from the asymptotic limit as *λ* increased. We therefore based our statistical tests in the presence of a non-vanishing penalizer on empirical null distributions of *q*_*ij*_ constructed by permutation resampling (Additional file 5: Figure S4).

We then sought to evaluate the sensitivity of causal interaction identification within different inference methods using simulations. We created simulated data of *m*=10 SNPs, this time with random parameters with mean values that were identical for both case and control groups, except a single SNP pair for which the case group had stronger interactions than the control (Fig. 2e–f). For collective inference (logistic regression and DDA), we first performed cross-validation for each sample to determine optimal *λ*^∗^, and then constructed the empirical null distribution under this *λ*^∗^ (Additional file 5: Figure S4) to calculate *p*-values of the causal and non-causal interaction pairs (Fig. 2e and f, respectively). We selected the simulation parameters such that the SNPs were fairly strongly coupled via LD in each of case and control groups, but these interaction effects were expected to cancel out except for the single causal pair. The pairwise test results remained insensitive to this causal interaction for all sample sizes. The logistic regression, DDA PL, and EE methods detected this interaction fairly robustly for *n*≥10^3^. In all cases, DDA had higher sensitivity than logistic regression. The *p*-values for the non-causal interactions mostly followed the expected null distribution qualitatively. However, the distributions from logistic regression were significantly broader (higher false positive rates; lower specificity) than DDA for all sample sizes.

In applications to actual disease data, where one aims to identify statistically significant pairs of interactions based on *p*-values, the enhanced sensitivity and specificity of detection shown in Fig. 2e are of more interest than the parameter prediction accuracy in Fig. 2a, c. Our results suggest that the sensitivity of detecting disease-associated interactions among mostly non-causal SNP pairs from noisy data is significantly higher with collective inference than with pairwise tests. The DDA inference furthermore allowed for consistently higher sensitivity and specificity than logistic regression.

### Age-related macular degeneration

**Independent-SNP** We first analyzed AMD data under the independent-SNP assumption and compared the logistic regression and DDA results. Analytic expressions are available for the odds ratio and *p*-values for DDA [Eqs. (S24), (S27) and (S28) in Additional file 1: Text S1]. Genome-wide *p*-values derived from DDA (Additional file 6: Figure S5) were consistent with published results [41]. The *p*-values from independent-SNP logistic regression using PLINK and those from DDA for three main associated genomic regions (*CFH*, *C2/CFB*, and *ARMS2* gene groups in chromosomes 1, 6, and 10, respectively) were the same for most loci except those with strongest associations, for which *p*-values from DDA were slightly smaller (Additional file 7: Figure S6). Differences in *p*-values were larger with the genotypic model than with the dominant model (Additional file 1: Table S1). Thus, when interactions are not included, DDA gives nearly the same results as the logistic regression inference. This feature allows one to directly interrogate how collective interactions modify association.

**Fig. 3:**
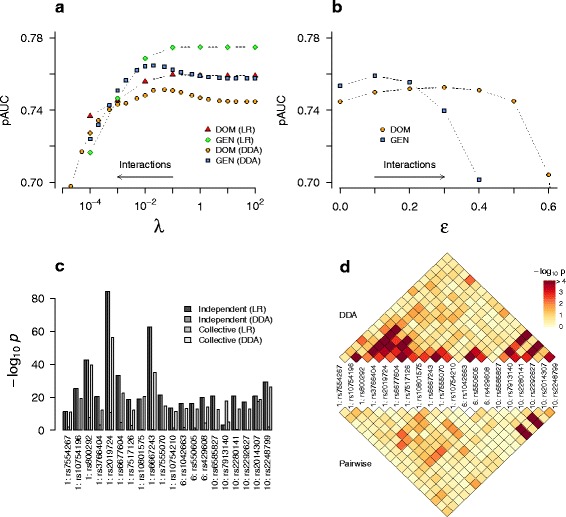
Collective inference applied to pre-selected *m*=20 AMD SNPs. **a**–**b** Regularization via cross-validation. Dominant (DOM) and genotypic (GEN) models were used with logistic regression (LR), DDA PL (**a**), and MF (**b**). Independent-SNP limit is reached with *λ*→*∞* and *ε*→0. Because of the pre-selection of SNPs using phenotype information, the prediction score (pseudo-AUC; pAUC) derived from 5-fold cross-validation over-estimates the true AUC. The maxima in pAUC correspond to optimal regularization. **c**–**d** Single-SNP and interaction *p*-values of the optimized (genotypic) model under PL (*λ*=0.01). The *p*-values from independent-SNP and pairwise tests are also shown for comparison in **c** and **d**, respectively. See Additional file 1: Table S1 for the independent-SNP results and SNP list

**Collective inference for 20 SNPs** We then examined the performance of DDA on AMD data under the first mode of application, where detailed interaction patterns within a relatively small set of pre-selected SNPs are inferred. We selected *m*=20 AMD SNPs using the variable selection program GWASelect [47] (see Additional file 1: Table S1), which covered most regions previously identified as strongly associated with AMD risks (Additional file 6: Figure S5 and Additional file 7: Figure S6). The independent-SNP *p*-values of this set are shown in Fig. 3c. For the majority of loci (18 out of 20), the risk allele was the major allele, and odds ratios were smaller than 1. As stated above, under this condition of no interaction, the *p*-values from logistic regression (from PLINK) and those from DDA (analytic) were nearly the same.

We applied collective inference (including interactions) to this 20-SNP set using logistic regression and DDA. We first performed cross-validation to determine the optimal penalizer *λ*^∗^ (Fig. 3a–b). It should be noted that because the pre-selection of SNPs in this case used phenotype information of the entire sample, the cross-validation prediction score is not an unbiased estimate of the true AUC and is generally higher in value [37]. In our application, this procedure merely allows for the identification of optimal regularization levels for collective inference. We denote the prediction score derived after such pre-selection using sample phenotypes as *pseudo*-AUC (pAUC) in order to distinguish it from the true estimate of AUC. Unbiased estimates of AUC, if desired, can be obtained, for example, by performing independent-SNP *p*-value-based filtering based only on training sets of each cross-validation sub-division [37] (see below) or by using selection criteria unrelated to sample phenotypes (e.g., pathways).

As observed with the simulated data, when DDA was used, the pAUC values with varying regularization levels showed a maximum (Fig. 3a–b), which corresponds to the optimal degree of interaction effects to be included in genotype distributions. For PL (Fig. 3a), the maxima were located at *λ*^∗^=0.05 (pAUC=0.751) and *λ*^∗^=0.02 (pAUC=0.765) for the dominant and genotypic models, respectively. The slightly higher pAUC suggests that for this data set, the genotypic model provides a better fit. For DDA, we verified that in the large- *λ* limit, the inference outcome approaches the independent-SNP result. The difference between this limit and the maximum pAUC is a measure of the relative importance of interactions in disease association.

We also applied logistic regression-based collective inference to the same data set. Cross-validation yielded similar differences between the dominant and genotypic models (Fig. 3a). However, pAUC did not exhibit pronounced maximum, instead approaching a large- *λ* limit nearly monotonically. This limit was slightly higher than the corresponding DDA maximum, which is consistent with the expectation that logistic regression can yield better prediction performance because it maximizes the prediction score [Eq. (8) in Methods]. On the other hand, the absence of pronounced maximum in pAUC as a function of *λ* indicates a loss in sensitivity in logistic regression for the detection of interaction effects. This conclusion can be rationalized in terms of the algorithmic difference between logistic regression and DDA: in DDA, case and control group genotype distributions are fit separately in terms of their respective single-SNP and interaction parameters, whereas logistic regression optimizes the prediction score with respect to the *net differences* in those parameters. With more flexibility to account for differential population structures, DDA has higher sensitivity to detect interaction effects.

Figure 3b shows the analogous model optimization under the DDA MF method, where regularization parameter values *ε*=0 and *ε*=1 each correspond to independent-SNP and full interaction limits, respectively. The maximal pAUC values from MF were similar but slightly lower in comparison to PL. On the other hand, MF is more computationally efficient that PL and allows for larger SNP sets.

We used the optimal penalizer value to determine the parameters and *p*-values for this 20-SNP data set under the genotypic model using DDA PL. The *p*-values, representing the statistical significance of the individual terms included in the model, consist of two classes: single-SNP and interactions. The single-SNP *p*-values represent the significance of marginal single-site effects (associated with parameters $h_{i}^{(y)}$ or *β*_*i*_). They are analogous to the independent-SNP *p*-values of each SNP, but having been inferred in the presence of interactions, they also indirectly reflect interaction effects. Strictly speaking, the presence of penalizer *λ* also affects the distribution of the likelihood ratio statistics *q*_*i*_ and it is desirable to estimate their *p*-values using permutation resampling. However, since we did not impose penalty to single-SNP terms directly [Eq. (4) in Methods], we expect this effect to be moderate. In practice, these *p*-values tend to be much smaller than 1 for SNPs selected based on independent-SNP properties, and they are difficult to estimate using resampling. We chose to use the asymptotic *χ*^2^-distribution to estimate these single-SNP *p*-values under collective inference. These are therefore expected to be upper-limits (i.e., actual *p*-values are expected to be smaller) based on the observation that the penalizer tends to suppress null distributions to lower *q*-region.

Figure 3c shows the collective inference single-SNP *p*-values of the *m*=20 AMD data from DDA. They largely retained the relative strengths of significance in independent-SNP results, while in absolute magnitudes the − log10*p* values were mostly reduced. This feature indicates that in comparison to the independent-SNP model where single-SNP parameters also contain average effects of interactions, when collectively inferred, these terms make reduced contributions to association because they represent single-site effects only. We also performed analogous calculations using logistic regression, adopting the lowest value of penalizer *λ*=0.1 at which pAUC reached the limiting value in Fig. 3a. The single-site *p*-values showed larger deviations from the independent-SNP results (Fig. 3c), with values for many sites becoming insignificant.

We then performed resamplings of this data set to obtain interaction *p*-values (Fig. 3d), which indicated strong interactions within the *CFH* gene group in chromosome 1, *C2* in chromosome 6, and *ARMS2/HTRA1* group in chromosome 10. In contrast, pairwise test *p*-values detected strong interactions only within the last group of loci, between rs6585827/rs2280141 and rs2014307/rs2248799 (*p*∼10^−9^). These short-range interactions suggested by DDA tended to be correlated with LD: because the net disease association is related to the difference in SNP correlation patterns between case and control groups (Fig. 1), we interpret these short-range interactions as the consequence of differential LD-patterns in case and control individuals. The absence of such signals in pairwise test *p*-values suggests that such differences get averaged out when represented only by marginal SNP-pair distributions.

**Fig. 4:**
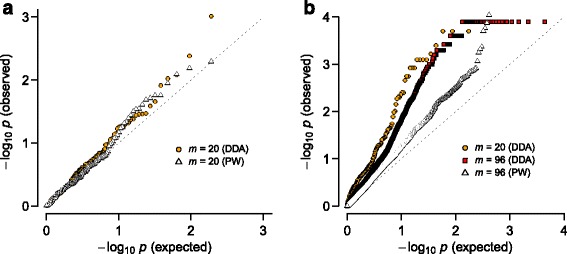
Quantile-quantile plot of interaction *p*-values. **a** Distributions for interactions among *m*=20 SNPs randomly selected from genome-wide data. **b** Distributions for interactions among 20 SNPs with high association (Fig. 3 d) and a larger set (*m*=96; Fig. 6). See Additional file 8: Figure S7 for pairwise (PW) results for *m*=96

We sought to further test whether such increased sensitivity toward interactions was achieved with adequate control for false discovery rates. The selection of *m*=20 SNPs in Fig. 3c–d comprises SNPs with highest association. For comparison, we made a random selection of *m*=20 SNPs from the genome-wide data and performed DDA as well as pairwise test. The quantile-quantile plot (Fig. 4a) showed that *p*-values for interactions between these random SNPs were distributed close to the null distribution. In particular, DDA and pairwise outcomes were similar, except for one pair for which DDA predicted *p*∼10^−3^. In contrast, the distribution of interaction *p*-values for the highly associated *m*=20 SNPs (Fig. 3) from DDA deviated significantly from the null (Fig. 4b), whereas the pairwise test outcome remained similar to random SNPs except for ∼10 SNP pairs. These results suggest that DDA achieves increased sensitivity for interactions while adequately controlling for false positive rates.

**Fig. 5:**
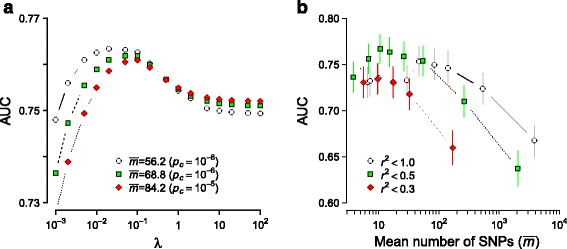
Collective inference with SNP selection based on independent-SNP *p*-values. **a** AUC with varying penalizer *λ* under PL inference, where independent-SNP *p*-value cutoff *p*_*c*_ indicated was used to filter SNPs from the full genome-wide set in each cross-validation run. The mean SNP number ${\bar m}$ is the average over 5 runs. **b** AUC optimized over regularization (MF) with varying model sizes controlled by *p*_*c*_. SNP selections were made from the full genome-wide data (*r* ^2^<1.0) and subsets generated by pruning based on LD thresholds indicated. Note that the maximum AUC position shifts to lower ${\bar m}$ with increasing degree of pruning (fewer SNPs with LD needed to account for association) and that an optimal level of pruning (*r* ^2^<0.5) exists for highest performance. Vertical lines are 95 % C.I

**Large-scale collective inference** The analysis described above used a fixed number of pre-selected SNPs to perform cross-validation and inferences. We next enlarged the size of SNP sets by controlling it using an independent-SNP *p*-value cutoff *p*_*c*_; with the cutoff specified, in each cross-validation run, the training set was used to obtain independent-SNP *p*-values, filter SNPs, and perform inferences, and the test set was used for prediction. The prediction score derived under this protocol is an unbiased estimate of the true AUC. The AUC values (Fig. 5a) showed qualitative trends similar to Fig. 3a; the AUC maximum relative to the non-interacting limit was more pronounced, while its height showed a moderate decrease with increasing SNP numbers: inclusion of less-significant SNPs diluted the overall effects. We chose a SNP-set size of *m*=96 (*p*_*c*_=10^−5^ without cross-validation) for interaction pattern analysis. The interaction *p*-value computation for *m* SNPs entails a multiplication of the single-inference computing time by *m*(*m*−1)/2 (the number of pairs) times the necessary random resampling size (∼10^3^ or more) for *p*-value estimation, thus limiting model sizes that can be considered to *m*∼100.

**Fig. 6:**
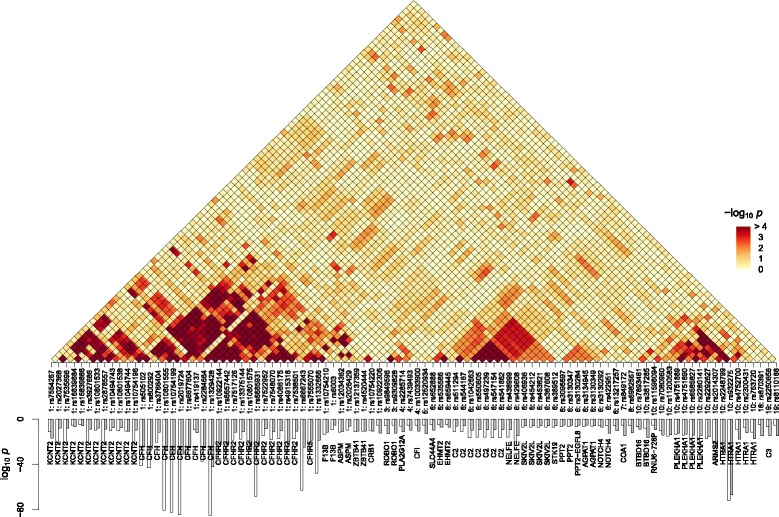
Interaction and single-site *p*-values for *m*=96 AMD SNPs. The bars (*bottom*) and the heat map (*top*) show the single-SNP and interaction *p*-values, respectively. *Hollow* and *solid bars* represent the independent-SNP and collective inference *p*-values respectively. DDA PL was used for collective inference

The resulting single-site and interaction *p*-values are shown in Fig. 6, where the bottom panel compares the independent-SNP/collective single-site *p*-values. As in Fig. 3d for the smaller SNP set, the collective single-site significance of strongly associated SNPs was generally reduced in strength compared to the non-interacting case. However, rs932275 in chromosome 10 had a comparable *p*-value (strongest within the collective inference results) and many SNPs originally with weaker associations in the non-interacting case became stronger under collective setting. The interaction landscape shown on the top panel of Fig. 6 retained the qualitative trend of the results from the smaller data set in Fig. 3d, but with much more detail; we confirmed the presence of local interactions within the *CFH*, *C2*, and *ARMS2* gene groups. In addition, we observed numerous ‘long-range’ interactions that were absent in the *m*=20 results: rs2284664 in *CFH* interactions with rs511294 and rs544167 in *C2*, and there were additional distributed interactions between the *CFH* loci and the *ARMS2* gene group. The distribution of interaction *p*-values was similar to that for *m*=20 in the quantile-quantile plot (Fig. 4b). The pairwise test *p*-value landscape for the same data (Additional file 8: Figure S7) was also qualitatively similar to the *m*=20 case (Fig. 3d, *bottom*).

The overall picture of disease-associated epistatic interactions from our small and larger-scale collective inferences in Figs. 3d and 6 provides a plausible explanation of the recent observation by Hemani et al. [31], who detected many epistatic SNP pairs leading to differential gene expressions within the human genome by exhaustive searches using pairwise tests. Wood et al. [48] then observed that many of these effects could be explained by single untyped third SNPs in LD with the interacting pairs. Here, we observed both from simulations and AMD SNP analyses that pairwise tests (Fig. 3d, *bottom* and Additional file 8: Figure S7) detect only a subset of statistically significant interactions, and a portion of the interaction patterns uncovered from collective inference parallels that of the underlying LD (Fig. 6 and Additional file 9: Figure S8): SNPs with strong overall correlations often also have differential LD between case and control groups. It is thus understandable that interacting pairs of SNPs identified in marginal pairwise tests turn out to be in LD with other hidden variants. Our results in Fig. 6, however, demonstrate a strong presence of interactions beyond both the population LD (Additional file 9: Figure S8) and the reach of pairwise tests (Additional file 8: Figure S7).

**Fig. 7:**
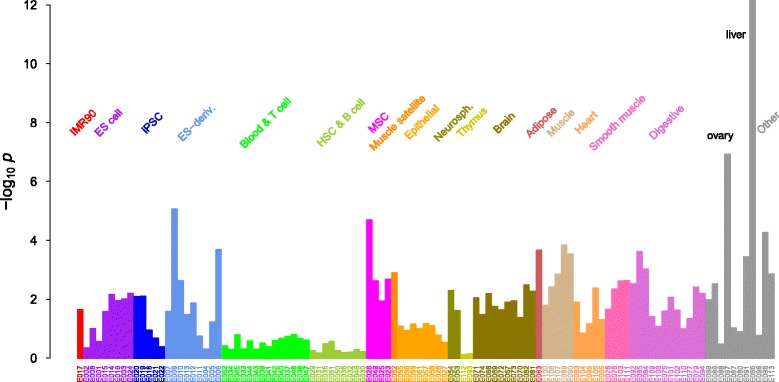
Enrichment *p*-values of active epigenetic states among AMD-associated SNPs. The set of 96 SNPs in Fig. 6 was used. The reference epigenome labels are as defined in Fig. 2 of Ref. [50]. ES, embryonic stem cell; ES-deriv., ES cell-derived; HSC, hematopoietic stem cell; iPS, induced pluripotent stem cell; MSC, mesenchymal stem cell; Neurosph., neurosphere

**Disease-associated epigenomes** Most of the disease-associated loci from GWAS reside in non-coding regions, presumably exerting their effects through modifications of gene regulatory action [49]. The overlap of epigenetic signatures with disease-associated SNPs and their interactions can provide important biological insights to the underlying disease mechanism. We sought to identify tissue and cell type-specific interaction patterns associated with AMD phenotypes using the SNP interaction map we derived above (Fig. 6). We used the recently published NIH Roadmap Epigenomics Consortium data [50] to first calculate the enrichment *p*-values of the transcribed/enhancer states among the selected 96 AMD SNPs within each of the 111 reference epigenomes (Fig. 7). We combined the actively transcribed and enhancer states of the 15 chromatin state annotations of the reference epigenomes to define the ‘active’ state. For each AMD-associated SNP, we identified the group of all known SNPs that were strongly correlated (high LD), obtained the distribution of epigenetic states over these SNPs within a given epigenome, and tested the over-representation of the active state against the background distribution. This enlarged search over the set of all known SNPs in LD with the locus included in inference is crucial to address the issue of the incomplete coverage of genotype data.

The most prominent feature in Fig. 7 is the strong enrichment of active epigenetic states among AMD SNPs within the liver tissue (E066), followed by ovary (E097). Two additional epigenomes, embryonic stem cell-derived neuronal progenitor cultured cells (E009) and bone marrow-derived mesenchymal stem cells (MSCs; E026), were also notable, but their enrichment *p*-values on the single-SNP level were comparable to other tissues.

We then hypothesized that the statistically significant interactions between SNPs identified in Fig. 6 would provide additional information of the cell-type specificity of epigenetically active states and their interactions. We selected the SNP pairs with interaction *p*<10^−3^ from Fig. 6 and, assuming that each groups of LD-correlated SNPs came from specific cell types (111×112/2 possible pairs, including self-interactions), tested the enrichment of active state pairs. The *p*-values derived then reflect the statistical significance of the epigenetic modifications enabling the interactions occurring between two cell types that are disease-associated.

**Fig. 8:**
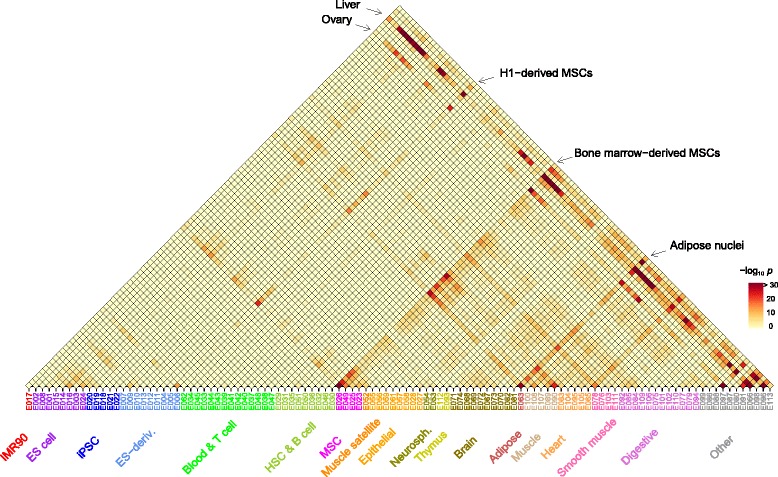
Enrichment *p*-values of active epigenetic state pairs among AMD-associated SNP interactions. The SNP pairs with interaction *p*-value <10^−3^ in Fig. 6 were tested for enrichment within each reference epigenome pairs

The resulting landscape shown in Fig. 8 exhibited strong interactions involving the liver tissue, consistent with the single-SNP result in Fig. 7. However, clear patterns not seen on the single-SNP level also emerged: bone-marrow derived MSCs (E026) and adipose nuclei (E063) also featured prominently in the interaction landscape; the bulk of interactions involving the liver tissue was accounted for by those with MSC, adipocytes, and muscle tissues. Embryonic stem cell H1-derived MSCs (E006) showed interactions that were weaker but similarly distributed in comparison to bone-marrow derived counterparts. The ovary followed patterns similar to the liver but was less pronounced than in Fig. 7. In addition, lung (E096) and placenta (E091) showed some interactions with adipocytes and MSCs. All of these tissues strongly interacted with themselves: interacting SNPs in these tissues are highly likely to be active epigenetically.

**SNP selection from genome-wide data** Collective inference without interaction *p*-value computation can be applied to SNP sets of sizes up to *m*∼10^4^. The prediction AUC as the main outcome for each SNP selection then allows for the comparison of the relative strengths of disease association of different SNP groups. In such applications, the performance of DDA could depend on (phenotype-independent) processing applied to genome-wide data in selecting SNP sets for analysis. We evaluated this second mode of DDA application and assessed how its performance varied depending on the degree of LD within SNP sets. We generated different subsets of genome-wide SNPs by pruning, removing variants that had LD with neighboring SNPs higher than a threshold (Fig. 5b). The AUC obtained with SNPs selected from the full genome-wide data peaked around the mean number of SNPs $\bar {m}~\sim 50$, as suggested also by Fig. 5a. With LD-based pruning, the position of maximum shifted to levels up to $\bar {m}\sim 10$, which suggests that about 10 SNPs in linkage equilibrium account for the bulk of the association. The height of the AUC first increased with the data pruned with *r*^2^<0.5 compared to the full set and then decreased with *r*^2^<0.3, indicating that there is an optimal level of pruning beyond which key causal SNPs begin to be removed. Overall, the relatively small model size ranges where collective inference performance is maximized in Fig. 5b suggests that AMD is only weakly polygenic with dominant contributions from a few loci. Analyses of the type demonstrated in Fig. 5b thus allows one to assess the polygenicity of the phenotype under consideration and choose suitable strategies for SNP selection.

**Fig. 9:**
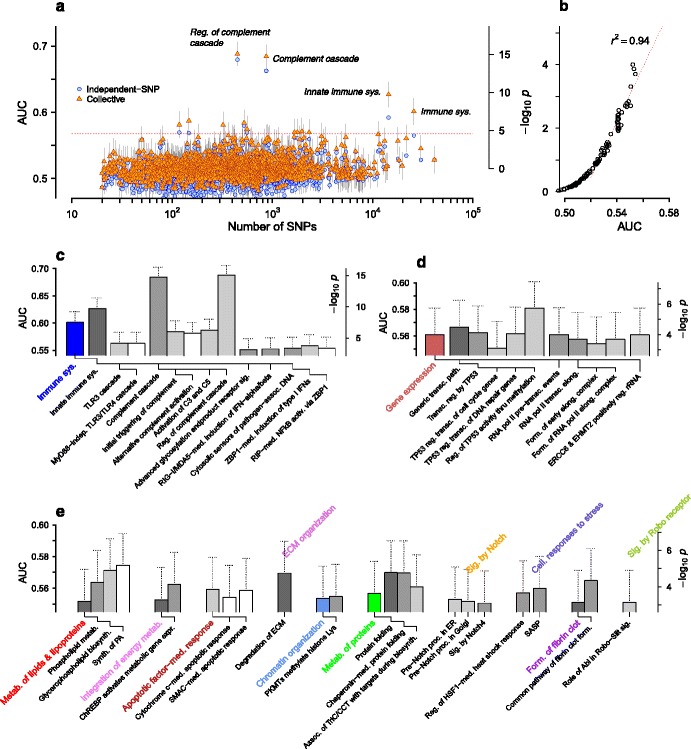
AMD association of pathways under collective inference. **a** AUC score versus pathway size (number of SNPs in each pathway). Symbols show collective and independent-SNP inference AUCs under 5-fold cross validation. Vertical lines are 95 % C.I. The horizontal line represents the Bonferroni-corrected nominal discovery threshold based on the *p*-value estimates. **b** Regression of AUC versus pathway *p*-values. The latter were obtained for a selection of pathways via phenotype-label reshuffling using AUC as the statistic. Dotted line is the linear fit for AUC>0.52. **c**–**e** Pathways with association strength AUC>0.55, grouped according to the top hierarchical classes they belong to. We excluded pathways in the *Disease* class. Dendrograms below the bars show their hierarchical relationships. Abl, Abl tyrosine kinase; activ., activation; assoc., association/associated; biosynth., biosynthesis; C3, complement component 3; C5, complement component 5; CCT, chaperonin-containing T-complex polypeptide 1; cell., cellular; ChREBP, carbohydrate response element-binding protein; ECM, extracellular matrix; EHMT2, euchromatic histone-lysine-methyltransferase 2; elong., elongation; ER, endoplasmic reticulum; ERCC6, excision repair cross-complementation group 6; expr., expression; form., formation; HSF, heat shock factor; IFN, interferon; indep., independent; Lys, lysine; MDA5, melanoma differentiation-associated gene 5; med., mediated; metab., metabolism; MYD88, myeloid differentiation primary response 88; NFKB, nuclear factor- *κ* B; PA, phosphatidic acid; PKMT, protein lysine methyltransferase; pol, polymerase; proc., processing; reg., regulate/regulation/regulated; RIG-I, retinoic acid-inducible gene-I; RIP, receptor-interaction protein; Robo, roundabout; SASP, senescence-associated secretory phenotype; sig., signaling; SMAC, second mitochondrial activator of caspases; synth., synthesis; sys., system; thru, through; TP53, tumor protein p53; transc., transcription/transcriptional; TriC, T-complex polypeptide 1 ring complex; ZBP1, Z-DNA-binding protein-1

**Pathway-based SNP selection** An obvious criterion for grouping genome-wide SNPs into subsets for collective inference-based evaluation is the proximity to gene sets belonging to known biological pathways. From the full AMD genome-wide data, we generated 1,732 SNP sets corresponding to 1,732 Reactome pathways [51], each containing from 20 to ∼10^4^ SNPs. We then applied DDA MF inference and derived optimized AUC values for each pathway (Fig. 9a). The majority of the pathways had association levels [median AUC: 0.514±0.021 (95 % C.I.)] close to the null value of 0.5. The differences in collective inference AUC relative to independent-SNP results ranged from 0 to ∼0.06. Reflecting the dominance of the complement-related genetic loci, *Regulation of complement cascade* (AUC: 0.688±0.018, *m*=448) and *Complement cascade* (AUC: 0.684±0.018, *m*=869) showed top association levels clearly separated from the rest. These AUC values were similar to the levels observed in *p*_*c*_-based sampling in Fig. 5b adjusted to their corresponding SNP numbers. We used a selection of pathways with low AUC values to sample their null distributions and connect AUC (as the statistic for each pathway) and *p*-values corresponding to the overall association of each SNP set (Fig. 9b). The − log10*p* values monotonically increased from 0 as AUC increased from 0.5, and became highly linear for AUC>0.52 (*r*^2^=0.94). We used this linear regression formula to translate AUC into *p*-values. The Bonferroni correction with 1,732 pathways to the nominal false discovery rate indicated a threshold of AUC>0.567, which led to 13 pathways above the threshold shown in Table 1.

**Table 1:** Pathways highly associated with AMD in collective inference

Rank	Pathway	No. of SNPs	AUC ^*a*^	*p*-value ^*b*^
1	Regulation of complement cascade	448	0.688 (0.018)	9×10^−16^
2	Complement cascade	869	0.684 (0.018)	2×10^−15^
3	Innate immune system	14, 406	0.627 (0.019)	2×10^−10^
4	Immune system	25, 770	0.601 (0.020)	3×10^−8^
5	Activation of C3 and C5	147	0.587 (0.020)	5×10^−7^
6	Initial triggering of complement	522	0.584 (0.020)	1×10^−6^
7	Alternative complement activation	118	0.581 (0.020)	2×10^−6^
8	Regulation of TP53 activity through methylation	322	0.581 (0.020)	2×10^−6^
9	Synthesis of phosphatidic acid	559	0.575 (0.020)	7×10^−6^
10	Glycerophospholipid biosynthesis	1, 969	0.571 (0.020)	1×10^−5^
11	Protein folding	1, 777	0.570 (0.020)	2×10^−5^
12	Chaperonin-mediated protein folding	1, 661	0.570 (0.020)	2×10^−5^
13	Degradation of extracellular matrix	2, 324	0.570 (0.020)	2×10^−5^

^a^95 % C.I. in parentheses

^b^Estimated from the regression in Fig. 9 b

C3, complement component 3

C5, complement component 5

TP53, tumor protein p53

**AMD disease mechanism** We sought to gain insights to molecular-level disease mechanisms of AMD by examining the pathways in Table 1 along with additional pathways near the threshold and grouping them into hierarchical classes (Fig. 9c–e). There are two primary types of AMD, the ‘dry’ and ‘wet’ forms [52]. The dry AMD more commonly occurs in earlier stages, where retinal pigment epithelium (RPE) cells supporting photoreceptors in the retina undergo degeneration, often accompanied by the accumulation of drusen in the area between RPE and the Bruch’s membrane separating the retina from the choroid. The wet AMD is characterized by invasive choroidal neovascularization of the retina. In both forms, cellular stress factors exacerbated by aging are the primary causes leading to RPE dysfunction. The normal functioning of photoreceptors, bombarded by light and highly susceptible to oxidative damage, relies on continual recycling of their spent outer segments via phagocytosis by RPE cells [53]. Peroxidation products of phospholipids, the key ingredients of photoreceptors, often end up as major components of drusen, and serve as damage-associated molecular patterns activating innate immune receptors, including toll-like receptors (TLRs) as well as complement factor H (CFH) [54]. The latter has been shown to bind malondialdehyde (MDA) derived from docosahexaenoic acid [55]. In addition, phosphatidylserine is the main ‘eat-me’ signal toward phagocytes when displayed on the extracellular membrane of dying cells [56]. Consistent with these aspects of AMD pathogenesis, we found associations with *Phospholipid metabolism* pathways (Fig. 9e), and in particular, *Synthesis of phosphatidic acid*, which suggests that disease risk is affected by genetic variants modifying the ability to supply these phospholipids.

Phospholipid synthesis requires the supply of fatty acids, synthesized in the liver. The causal link to this process of lipogenesis is suggested in Fig. 9e by the pathway *Carbohydrate response element-binding protein (ChREBP) activates metabolic gene expression*. ChREBP is a key transcription factor in hepatocytes, responding to glucose uptake and activating genes involved in lipogenesis [57, 58]. Fatty acids thus synthesized are transported into the bloodstream in the form of very low density lipoproteins and stored as triacylglycerols in adipocytes [57]. The suggested AMD risk association of the fatty acid supply from the liver and adipocytes for phospholipid synthesis provides an explanation of our earlier finding in Fig. 8 that SNP interactions associated with AMD are epigenetically active in the liver and adipocytes.

Oxidative stress is often accompanied by disruptions to protein folding, which can lead to protein aggregation and autophagy when refolding by chaperones proves inadequate [59]. We found association in *Protein folding* pathways (Fig. 9e), and in particular *Chaperonin-mediated protein folding*, which primarily targets actins and tubulins, the major ingredients of cytoskeletal networks [60]. This observation suggests that RPE stress from protein misfolding affects AMD risk via its effect on phagocytic function, which relies heavily on actin filament and microtubule remodeling dynamics [56]. Also closely related is the *Regulation of heat shock factor (HSF) 1-mediated heat shock response* in Fig. 9e, which describes the transcriptional activation of heat shock protein (chaperone) expression under stress. The latter pathway belongs to the *Cellular responses to stress* group, in which we also found association with *Senescence-associated secretory phenotype* (SASP). Senescence is one of the possible fates of cells under stress, where normal cellular growth is arrested in preparation for clearance by phagocytes [61]. SASP refers to a complex suite of inflammatory cytokines, chemokines, and growth factors facilitating the process, and we infer that senescence in RPE cells under oxidative stress plays a part in AMD.

Apoptosis, or controlled cell death [62], is another major stressed-cell response, and was also represented in our results (Fig. 9e). A large body of direct evidence points to apoptosis as one of the main routes of RPE degeneration in AMD [63]. Induction of apoptosis upon stress is dictated by the action of master regulator p53, and it was recently shown that aging increases the activity of p53 in RPE cells and the likelihood for apoptotic cell death [64]. Consistent with this evidence, we found association with pathways in *Transcriptional regulation by TP53* group (Fig. 9d). In particular, *Regulation of TP53 activity through methylation* was among the top pathway in our association analysis (Table 1), suggesting that p53 modification by methylation and the closely related histone modifications [*Protein lysine methyltransferases (PKMTs) methylate histone lysine* in Fig. 9e] play important roles in RPE apoptosis regulation. In the intrinsic apoptotic pathway induced by oxidative stress, cytochrome c is released from mitochondria into the cytosol, binding and activating caspases, the main proteases central to apoptotic action. We found association in pathways involving ‘inhibitor of apoptosis’ (IAP) and its negative regulator ‘second mitochondrial activator of caspases’ (SMAC) [65], which suggests that disruption to regulatory mechanisms preventing apoptosis in RPE cells may play roles in AMD.

RPE degeneration and drusen formation can lead to inflammation, the main innate immune response involving a wide range of pattern-recognition receptors (PRRs) and complement activation [66]. Most of known PRRs were represented in our results (Fig. 9c), including TLRs, advanced glycosylation endproduct receptors, RIG-I-like receptors, and cytosolic DNA sensors [66]. Complement factors constitute the soluble counterparts of PRRs, and *Regulation of complement cascade* showed the highest association due to the contribution of CFH, as well as *Activation of C3 and C5*. CFH normally protects self tissues from complement-induced destruction by binding to a range of surface signals including glycoproteins and C-reactive protein. In addition to the binding of CFH to MDA noted above, it was also reported that CFH inhibits lipoprotein binding toward Bruch’s membrane [67]. The breach of Bruch’s membrane and the intrusion of blood vessels into the retina are the hallmarks of wet AMD [52], which are consistent with our finding of high association in *Degradation of extracellular matrix* and *Common pathway of fibrin clot formation* in Fig. 9e.

## Conclusions

In this paper, we first described and tested discriminant analysis-based algorithms inferring collective disease association effects applied to intermediate-sized SNP sets. Using simulated and actual disease data, we provided evidence suggesting that collective inference methods outperform pairwise tests and logistic regression in incorporating interaction effects in disease association.

We demonstrated two different modes of applying DDA in the analysis of actual disease data: one in which detailed interaction patterns within a relatively small set of SNPs are inferred, and the other where genome-wide SNP data are grouped into different subsets of SNPs and collective inference is used to compute the degrees of disease association of each subset. Our results applied to AMD in Fig. 9 based on pathway-based SNP selection, in particular, show that the latter protocol allows us to identify pathways encompassing a large fraction of disease mechanisms previously established by non-genetic means. Based on current study, we propose the following approach to deal with novel GWAS case-control data using DDA: first, characterize the degree of polygenicity of the data set with independent-SNP and collective inferences employing *p*_*c*_-based SNP selection and optimize the density of SNPs using LD-based pruning (Fig. 5). Second, classify SNPs into pathway-based groups, score them using collective inference, and seek insights to the underlying disease mechanisms by analyzing the results within the pathway hierarchy.

## Methods

### Genotype distribution of case-control groups

Our algorithm is best understood in comparison to the classical continuous variable discriminant analysis. Table 2 outlines the similarities and differences of classical (continuous variable) and discrete (our adaptation) versions of discriminant analyses. In the continuous variable case, one aims to classify data into two known groups, case and control (denoted by *y*=1 and *y*=0, respectively), based on predictor **a**, a vector of dimension *m*. Classification (and inference) are performed by maximizing the likelihood of model parameters given the training data of known class identities, i.e., the joint probability

1 $$ \text{Pr}(\textbf{a},y)=\text{Pr}(\textbf{a}|y)p_{y},   $$

**Table 2:** Comparison of continuous-variable/discrete discriminant analyses

Predictor, **a**	Classes	Parameters	Predictor distribution for class *y*	Prediction
Continuous variables	Case (*y*=1)	*μ*_1_, *Σ*_1_	*N*(*μ*_*y*_,*Σ*_*y*_)	Decision boundary:
	Control (*y*=0)	*μ*_0_, *Σ*_0_		*δ*_0_(**a**)=*δ*_1_(**a**)
Discrete variables	Case (*y*=1)	$\psi _{1}=\{h_{i}^{(1)},J_{ij}^{(1)}\}$	Pr(**a**;*ψ*_*y*_|*y*)	$\beta _{i}=h_{i}^{(1)}-h_{i}^{(0)}$
	Control (*y*=0)	$\psi _{0}=\{h_{i}^{(0)},J_{ij}^{(0)}\}$	given by Eq. (3)	$\gamma _{ij}=J_{ij}^{(1)}-J_{ij}^{(0)}$

where *p*_*y*_ is the marginal probability of group membership. One then finds the class-specific mean vectors *μ*_*y*_ and covariance matrices *Σ*_*y*_. These quantities define the predictor distribution within each class, which are assumed to follow a multivariate normal distribution: **a**∼*N*(*μ*_*y*_,*Σ*_*y*_), or

2 $$ \text{Pr}(\textbf{a}|y) \propto \exp\left({\mu_{y}^{t}} \Sigma_{y}^{-1} \textbf{a}-\textbf{a}^{t}\Sigma_{y}^{-1}\textbf{a}/2\right),   $$

where the superscript *t* denotes transpose. In this formulation, the maximum likelihood condition for Eq. (1) is equivalent to maximizing the discriminant function *δ*_*y*_(**a**) given by the exponent of Eq. (2) plus ln*p*_*y*_, which is used to classify an arbitrary data **a** into case if *δ*_1_(**a**)>*δ*_0_(**a**) and control otherwise [37]. It is also useful to note that if we assume that **a** is a scalar, this framework reduces to *t*-tests for the null hypothesis *μ*_0_=*μ*_1_.

For our application, the predictor **a** is the collection of genotypes, which is discrete. The description here applies to the binary model (dominant or recessive), such that *a*_*i*_=0,1 represent aa and Aa/AA for SNP *i* for the dominant model, and aa/Aa and AA for the recessive model (see Additional file 1: Text S1 for the genotypic model). Figure 1 illustrates the general spirit of the DDA algorithm. Training data of known phenotypes can be used to obtain allele frequency vectors and covariance matrices with elements ${\hat f}_{i}^{(y)}$ and ${\hat f}_{ij}^{(y)}$, respectively, where *i*,*j*=1,⋯,*m* are SNP indices. Note that these quantities are the exact counterparts of the continuous variable mean *μ*_*y*_ and covariance *Σ*_*y*_. We model the genotype distribution within each class in a form analogous to Eq. (2) [68]:

3 $$ \text{Pr}(\textbf{a}|y)\propto\exp\left(\sum_{i} h_{i}^{(y)}a_{i}+\sum_{i<j} J_{ij}^{(y)}a_{i} a_{j}\right),   $$

where $h_{i}^{(y)}$ and $J_{ij}^{(y)}$ are model parameters of the distribution that we refer to as single-SNP and interaction parameters, respectively. Comparing Eqs. (2) and (3), one can observe that these parameters $\psi _{y}\equiv \{h_{i}^{(y)},J_{ij}^{(y)}\}$, each multiplying predictor **a** in linear and quadratic fashion, respectively, are expected to be related to frequencies ${\hat f}_{i}^{(y)}$ and ${\hat f}_{ij}^{(y)}$. In contrast to the continuous case, however, the exact form of this relationship is unknown due to the discrete nature of **a**, except for the special case of independent SNPs (see Section S1.5 in Additional file 1: Text S1; we refer to the special case of *no interaction* as the independent-SNP case).

The inference of this relationship is the major computational component of DDA, and is based on maximizing the log-likelihood (*L*_*y*_) per individual,

4 $$ L_{y}/n_{y}=\frac{1}{n_{y}}\sum_{k\in y}\ln \text{Pr}\left(\textbf{a}^{k}|y\right)-\frac{\lambda}{2}\sum_{i<j} \left(J_{ij}^{(y)}\right)^{2},   $$

where the first summation is over genotype data of *n*_*y*_ individuals in group *y*, and *λ* denotes a regularization parameter (penalizer) that controls the contribution of the SNP interactions in comparison to the independent-SNP case. The independent-SNP limit is reached with *λ*→*∞*, where optimal values of $J_{ij}^{(y)}$ all become zero due to high penalty. We opted for an *l*_2_-penalizer rather than *l*_1_; the latter generally exerts stronger effects [69] but *l*_2_ is analytic and facilitates non-linear optimization. In Additional file 1: Text S1, we show implementations of three possible ways to perform this inference of varying computational cost and reliability: exact enumeration (EE), mean field (MF) [68], and pseudo-likelihood (PL) [70, 71] methods. The EE is essentially exact, but requires enumerations of all possible genotypes, and can only be used for *m*∼25 or less. We used this option to assess the reliability of the other two methods. Both MF and PL are approximate and can be used for *m*∼10^3^ or larger. The MF option involves matrix inversion and requires a different regularization: instead of *λ*, we used *ε*∈[ 0,1], where *ε*=0 corresponds to the independent-SNP limit with no interaction. The PL method uses *λ*>0 and has the advantage that it can be easily parallelized. We implemented parallel computations of PL using the message passing interface protocol.

### Disease risk

Once genotype distributions of case, control, and pooled (whole sample) groups have been inferred, Bayes’ theorem allows one to obtain disease risk:

5 $$ {}\text{Pr}(y\,=\,1|\textbf{a})\,=\, \frac{\text{Pr}\left(\textbf{a}|y=1\right)p_{1}}{\sum_{y^{\prime}}\text{Pr}\left(\textbf{a}|y^{\prime}\right)p_{y^{\prime}}} \,=\,\frac{1}{1+e^{-\alpha-\sum_{i}\beta_{i} a_{i}-\sum_{i<j} \gamma_{ij} a_{i} a_{j}}}.   $$

One can show that (Additional file 1: Text S1)

6a $$\begin{array}{@{}rcl@{}} \beta_{i}&=& h_{i}^{(1)}-h_{i}^{(0)}, \end{array} $$

6b $$\begin{array}{@{}rcl@{}} \gamma_{ij}&=& J_{ij}^{(1)}-J_{ij}^{(0)}. \end{array} $$

In other words, the single-SNP and interaction disease risk parameters *θ*={*β*_*i*_,*γ*_*ij*_} are given by differences in genotype distribution parameters between case and control groups. In addition, the overall likelihood ratio statistic is given by the sum of *L*_*y*_ subtracted by the pooled value (see Fig. 1). The parameter *α* is related to disease prevalence *p*_1_=1−*p*_0_ (see Additional file 1: Text S1).

We used cross-validation to determine the penalizer *λ* in Eq. (4). We first formed five training and test sets (of 4:1 size ratios) from the data and used the training set to select variants with independent SNP *p*-values below a cutoff. We calculated the AUC for different *λ* values and found an optimal choice. Even when the actual AUC values obtained are not high enough for a reasonable risk prediction, this procedure still allows us to identify optimal ranges of the model size (the role of interactions).

We used disease prevalence *p*_1_=*n*_1_/*n* for cross-validation because the training and test sets have the same sampling biases. In predicting risks with a prospective sample, however, this probability would have to be adjusted to known population phenotype frequencies. We implemented a software feature such that the disease prevalence, which affects the disease risk parameter *α*, can be re-specified when a parameter set inferred from case-control data is applied to an independent test set.

### Logistic regression

In contrast to DDA outlined above, the logistic regression uses

7 $$ {}\text{Pr}(\textbf{a},\!y)\,=\,\text{Pr}(y|\textbf{a})\text{Pr}(\textbf{a})\!\simeq\! \text{Pr}(y|\textbf{a}) \!\equiv\! \frac{1}{1\,+\,e^{-\alpha-\sum_{i}\!\beta_{i} a_{i}-\!\sum_{i<j} \gamma_{ij} a_{i} a_{j}}}  $$

instead of Eq. (1), assuming that the marginal genotype distribution is uniform. The parameters *α*,*β*_*i*_, and *γ*_*ij*_ are then directly determined by maximizing the likelihood of Pr(*y*|**a**) only:

8 $$ L/n=\frac{1}{n}\sum_{k}\ln \text{Pr}\left(y^{k}|\textbf{a}^{k}\right)-\frac{\lambda}{2}\sum_{i<j} \gamma_{ij}^{2},   $$

where *n*=*n*_0_+*n*_1_, with respect to *α*, *β*_*i*_, and *γ*_*ij*_. In general, these disease risk parameter values from logistic regression are different from those obtained via genotype distribution parameters *ψ*_*y*_ in DDA with Eq. (6); the latter contains the effects of the nonuniform marginal genotype distribution Pr(**a**) of the sample, while logistic regression does not. For comparison, we also implemented this multivariate logistic regression with an *l*_2_-penalizer. The logistic regression can yield higher prediction AUC than DDA because by maximizing Eq. (8), one optimizes prediction directly. However, the quantity maximized in DDA given by Eq. (4) (or in fact the sum *L*_0_+*L*_1_; see Additional file 1: Text S1), rather than the prediction score, is the true likelihood.

### Significance tests

We performed likelihood ratio tests to assess the statistical significance of the overall collective inference and individual loci/interactions. The *p*-values derived are conditional to the number of loci *m* and penalizer value *λ* (determined from cross-validation). The statistic was obtained by adding the log-likelihood values of case and control groups and subtracting that of the pooled group (see Text S1). We tested the significance of the single-locus contribution to disease association from site *i* by calculating the likelihoods $L_{y}[\!h_{i}^{(y)}=h_{i}]$, where the single-SNP parameters of site *i* were prescribed as their pooled values (restricted model), and evaluating the differences against the likelihood of the full model (all parameters optimized without restriction). Analogous tests were performed for SNP pair *i*,*j* with $L_{y}[\!J_{ij}^{(y)}=J_{ij}]$. The restricted model corresponds to the null hypothesis that the parameters belonging to a particular locus or interaction in case and control groups are the same as for those in the pooled group. For interaction statistics, we used the approach of constructing the empirical null distribution of the statistics under a given *λ* by permutation resampling: the phenotype data of a given sample with a certain penalizer *λ* value was randomly reshuffled to obtain realizations of the likelihood ratio statistics. This sampling was repeated multiple times (up to ∼10^4^) to construct empirical cumulative distribution functions of the statistic for each site, or SNP-pair, from which the *p*-values were estimated. For the single-locus contribution statistics, we calculated *p*-values using the asymptotic *χ*^2^-distribution.

### Simulation

In testing the inference algorithms using simulated data, samples of case-control genotypes containing *m*=10 or 20 loci and *n*=2*n*_0_ individuals were generated under randomly assigned parameters {*ψ*_0_,*ψ*_1_}. The model parameters were chosen with $h_{i}^{(y)}\sim N({\bar h}_{y},{\sigma _{h}^{2}})$, and $J_{ij}^{(y)}\sim N({\bar J}_{y},{\sigma _{J}^{2}})$. To generate simulated data from these distributions, we evaluated summations over all (2^*m*^ for binary models) possible genotypes to calculate their probability distribution using Eq. (3). For a given sample, cross-validation was first performed with *λ* values ranging from 10^−4^ to 10^2^ to determine the optimal value *λ*^∗^ that maximizes AUC. The parameters *θ* were then derived using the full sample and *λ*^∗^. For DDA, the single-SNP and interaction parameters for case and control groups (*ψ*_0_ and *ψ*_1_, respectively) were obtained and used to derive *θ*. In contrast, in logistic regression, *θ* was obtained directly. The mean square error was calculated for inferred *θ* relative to true values of all distinct single-SNP and interaction parameters. We performed different inferences using a common set of data for each realization of parameters. The mean square error was then averaged over 100 realizations of parameters. We also compared pairwise test results using PLINK [44] epistasis module. We used PLINK version 1.9 with logistic regression and either dominant or genotypic coding options.

### Age-related macular degeneration data

We obtained AMD case-control genotype data from the National Eye Institute Study of AMD Database (dbGaP accession number phs000684.v1.p1), which consisted of 2, 159 case and 1, 150 control individuals. Autosomal SNPs were filtered with the criteria of MAF >0.01, Hardy-Weinberg equilibrium *p*-value >10^−6^, and genotyping rate >0.05 [41] to yield 324, 713 SNPs in total. Independent-SNP DDA analyses were performed using Eq. (S24) and (S27) in Additional file 1: Text S1 and compared with logistic regression results from PLINK [44] in addition to our numerical logistic regression implementation. For each SNP, the minor allele was identified from the allele frequencies over the pooled sample.

Except otherwise stated, inferences on AMD data used the genotypic model. In all cases, *λ* (or *ε* for MF) was first determined from cross-validation (optimal value *λ*^∗^ with the maximum AUC) and later used consistently for parameter estimation and *p*-value calculation. Interaction *p*-values were obtained for a given SNP selection and *λ*^∗^ by resampling.

To generate SNP sets with reduced LD for *p*_*c*_-based selection (Fig. 5b), we used the pairwise LD-based pruning option of PLINK with window size of 50 kb and 5 SNPs for shifts along with *r*^2^-thresholds of 0.5 and 0.3. The two threshold choices yielded SNP sets with *m*=180,354 and 117,976, respectively.

In performing the epigenetic state enrichment analysis, for each SNP considered, we used the 1000 Genomes reference haplotypes [72] of European individuals to build the list of correlated SNPs (LD *r*^2^>0.5). We then used the NIH Roadmap reference epigenome chromatin state annotations [50] to construct the distribution of epigenetic states within each group of LD-correlated SNPs. We used the hidden Markov model-based 15 state-annotations of 111 reference epigenomes, selecting 8 states [active transcription start site (TSS), flanking active TSS, transcription at gene 5’ and 3’, strong transcription, weak transcription, zinc finger-associated, genic enhancers, and enhancers] to define the ‘active’ state, which contained the transcribed, promoter, and enhancer regions. For each SNP location, we calculated the fraction of LD-correlated locations in active states within each cell type. This fraction was summed over the list of associated SNPs (*m*=96 in Fig. 7) to give the effective number of active states observed, and compared with the background active state frequency estimated over the whole genome for each epigenome. The over-representation *p*-values were calculated by the binomial test.

Analogous calculations were performed for the SNP interaction enrichment analysis. We first selected statistically significant SNP pairs with *p*<10^−3^ from Fig. 6 (310 in total). We then considered each unique combination of two epigenomes (including self-interactions) and, for each SNP pair selected, obtained the fraction of active state-active state pairs with the two groups of LD-correlated SNPs assumed to belong to the two epigenomes. This fraction was summed over the list of SNP pairs and compared with the background expected pair number (product of background active state frequencies from two tissues). Over-representation *p*-values were calculated using the binomial test.

Pathway-based SNP sets were generated for human pathways in Reactome database [51]. We compiled the list of all genes, assigned SNPs in the AMD genome-wide set within 50 kb of the coding region to each gene, and collected SNPs corresponding to the gene set belonging to each pathway. Only those pathways with 20 or more SNPs were considered (1,732 in total). For most pathways with *m*<6×10^3^, DDA independent-SNP and collective inference (*ε*-optimized MF) inferences were applied to each SNP set without further filtering to derive 5-fold cross-validation AUC. For larger pathways, *p*_*c*_-based filtering was incorporated into cross-validation to reduce the model sizes.

## Abbreviations

AMD, age-related macular degeneration; AUC, area under the curve; CFH, complement factor H; ChREBP, carbohydrate response element-binding protein; DDA, discrete discriminant analysis; EE, exact enumeration; GeDI, genotype distribution-based inference; GWAS, genome-wide association study; LD, linkage disequilibrium; MDA, malondialdehyde; MF, mean field; MSC, mesenchymal stem cell; pAUC, pseudo-AUC; PL, pseudo-likelihood; PRR, pattern-recognition receptor; RPE, retina pigment epithelium; SASP, senescence-associated secretory phenotype; SNP, single-nucleotide polymorphism; TLR, toll-like receptor; TSS, transcription start site

## Additional files

Additional file 1 Supplementary material. **Text S1.** Mathematical formulation of inference algorithms. **Table S1.** Independent-SNP inference comparison of logistic regression (from PLINK) and DDA (GeDI). (PDF 283 kb)

Additional file 2
**Figure S1.** Inference properties of a single independent SNP. Log odds ratio (OR) and power (level of significance 0.05) inferred from case-control data of size *n*=2*n*_0_=2*n*_1_ using DDA (analytic) and logistic regression (numerical) are shown for the dominant model. Equation (S29) of Text S1 was used. The minor allele frequency *ϕ*_*y*_ for control and case groups were set such that $f^{(y)}=2\phi _{y}(1-\phi _{y})+{\phi _{y}^{2}}=\phi _{y}(2-\phi _{y})$. We used *ϕ*_*y*_=(0.1,0.25) such that *f* ^(*y*)^=(0.19,0.4375) and *h* ^(*y*)^=(−1.45,−0.25) for control and case groups, respectively, and *β*=1.1987. (PDF 6.99 kb)

Additional file 3 **Figure S2.** Examples of true versus inferred parameters. a Dominant model with *m*=10 SNPs and inference on a sample of size *n*=10^3^. b Genotypic model with *m*=10 SNPs and inference on a sample of size *n*=10^5^. In all cases, the penalizer value was determined by cross-validation. (PDF 18.1 kb)

Additional file 4 **Figure S3.** Determination of penalizer *λ* via cross-validation. The data set is one realization of simulations shown in Fig. 2 b and the inference is with the exact enumeration (EE) method. The minima in mean square error (a) and the maxima in AUC (b) shift to lower *λ* as sample size *n* increases. (PDF 6.17 kb)

Additional file 5 **Figure S4.** Distributions of interaction likelihood ratio statistics under the null hypothesis. Empirical cumulative distribution functions (CDF) in terms of the interaction statistics *q* were obtained by resampling. Simulation conditions were as described in Fig. 2 e and inferences used EE. (PDF 7.39 kb)

Additional file 6 **Figure S5.** Whole-genome *p*-value profile of AMD data. Independent-SNP DDA with genotypic model was used. (PDF 25.7 kb)

Additional file 7 **Figure S6.** Regional views of AMD data. Independent-SNP DDA results (light blue) are compared to logistic regression from PLINK (yellow). Genotypic model was used. (PDF 10.9 kb)

Additional file 8 **Figure S7.** Marginal pairwise interaction *p*-values. PLINK epistatic module was used to *m*=96 AMD SNPs. SNP pairs with strongest *p*-values near *HTRA1* have *p*∼10^−9^. Genotypic model was used. The bottom panel shows the independent-SNP *p*-values. (PDF 54.6 kb)

Additional file 9 **Figure S8.** Linkage disequilibrium *r* ^2^ within *m*=96 AMD SNPs from PLINK. (PDF 48.3 kb)
