# Erratum to: Genotype distribution-based inference of collective effects in genome-wide association studies: insights to age-related macular degeneration disease mechanism

**DOI:** 10.1186/s12864-016-3095-2

**Published:** 2016-09-22

**Authors:** Hyung Jun Woo, Chenggang Yu, Kamal Kumar, Bert Gold, Jaques Reifman

**Affiliations:** 1 Biotechnology High Performance Computing Software Applications Institute, Telemedicine and Advanced Technology Research Center, U.S. Army Medical Research and Materiel Command, Fort Detrick, Maryland USA; 2 Laboratory of Genomic Diversity, National Cancer Institute, Frederick, Maryland USA

## Erratum

During the publication process, the published version of the original article [1] had a duplicate image of Additional File 3 in place of Fig. 2. The graphics for Fig. 2 have now been restored in the original article [1].

**Fig. 2:**
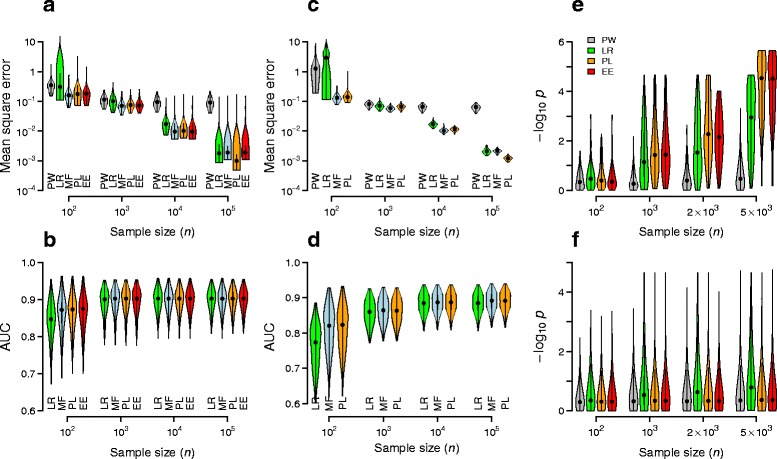
Inference accuracy, sensitivity, and specificity of pairwise and collective inference on simulated data. a–b The mean square error and AUC versus sample sizes using pairwise test (PW), logistic regression (LR), and the three methods of DDA (MF, PL, and EE). Simulated genotypes were generated for 10 SNPs with parameters *h*¯_*y*_ = (−1, −0.3), ¯ *J* = (0, 0.1), σ _*h*_ = σ _*J*_ = 0.2 (see Methods). c-d Analogous results for 20 SNPs with *h*¯_*y*_ = (−1, −1 + *h*), ¯ *J* = (0, *J*), and σ *h* = σ *J* = 0.2. We set *h* = 0.7, *J* = 0.5 for the first 4 SNPs and their interactions and *h* = *J* = 0 otherwise. e-f Sensitivity and specificity of disease-associated interaction pairs. Simulated data were generated with parameters *h*¯ = (−1, −1), ¯ *J* = (0.01, 0.01), σh = 0.1, σJ = 0.05 for m = 10 SNPs, except the interaction between the first two SNPs, for which we set ¯ *J* = (0.01, 0.11). Interaction p-values for all pairs were calculated either by PW or by regularization to determine λ∗ followed by the construction of null distribution under λ∗ (Additional file 5: Figure S4) for LR, PL, and EE. The distribution of p-values for the true causal interaction pair and those of non-causal pair (geometric mean) are shown in e and f, respectively. The dominant model was used in all cases
