# Supplementary material for: Genotype distribution-based inference of collective effects in genome-wide association studies: insights to age-related macular degeneration disease mechanism
Source: BMC Genomics. 2016 Aug 30;17(1):695. doi: 10.1186/s12864-016-2871-3 (PMC5006276; doi:10.1186/s12864-016-2871-3)
Supplement: Supplementary file 1 — Supplementary material. Text S1. Mathematical formulation of inference algorithms. Table S1. Independent-SNP inference comparison of logistic regression (from PLINK) and DDA (GeDI). (PDF 283 kb) [file 12864_2016_2871_MOESM1_ESM.pdf]

Supplementary Material

Genotype distribution-based inference of collective effects in genome-wide association studies: Insights to age-related macular degeneration disease mechanism

Hyung Jun Woo, Chenggang Yu, Kamal Kumar, Bert Gold, and Jaques Reifman

S1 Supplementary Text

In this section, we provide details of the method presented in the main text, also generalizing to $1 + Y$ different classes ($y = 0, \cdots, Y$, each representing control and phenotype groups of different disease severity; $Y = 1$ gives the case-control scheme) and the genotypic model, where $a_i = 0, 1, 2$ represents aa, Aa, and AA alleles; we write $a_i = 0, \cdots, l$ for the general case such that $l = 1$ for binary (dominant/recessive) models and $l = 2$ for the genotypic model.

S1.1 Likelihood function

The overall log likelihood of the model given the data is

$$L = \sum_{k=1}^{n} \ln \Pr(\mathbf{a}^k, y_k) = \sum_{y=0}^{Y} \sum_{k \in y} \ln \Pr(\mathbf{a}^k, y), \tag{S1}$$

where $\Pr(\mathbf{a}, y)$ is the genotype-phenotype joint probability and the last summation is over all individuals in category y. Introducing genotype distributions conditional to phenotypes

$$\Pr(\mathbf{a}|y) = \Pr(\mathbf{a}, y)/p_y, \tag{S2}$$

where p_y is the marginal probability of phenotype y, we have

$$L = \sum_{y} \sum_{k \in y} \ln \Pr(\mathbf{a}^k|y) + A \equiv \sum_{y} L_y + A, \tag{S3}$$

where $A = \sum_y n_y \ln p_y$. This likelihood function can be maximized with respect to p_y under the constraint $\sum_y p_y = 1$ using a Lagrange multiplier Λ:

$$0 = \frac{\partial}{\partial p_y} \left(L + \Lambda \sum_{y} p_y \right) = \frac{\partial A}{\partial p_y} + \Lambda = \frac{n_y}{p_y} + \Lambda, \tag{S4}$$

which yields $p_y = -n_y/\Lambda$. Using $\sum_y p_y = 1$, $\Lambda = -n$ and $p_y = n_y/n$. Since A is constant, the total likelihood of joint probability is a sum over those for each phenotype groups.

S1.2 Genotype distributions

As explained in the Methods Section of the main text, the genotype distribution $\Pr(\mathbf{a}|y)$ of group y can be modeled with the set of parameters $\psi_y = \{h_i^{(y)}(a), J_{ij}^{(y)}(a,b)\}$, each representing the single-SNP and

interaction contributions to the distribution. They are analogous to the linear and quadratic coefficients multiplying the predictor variable in the exponent of the normal distribution [Eq. (1) in the main text].

The genotype distribution is written as

$$\Pr(\mathbf{a}|y) = \frac{e^{H_y(\mathbf{a})}}{Z_y}, \tag{S5}$$

where

$$H_y(\mathbf{a}) = \sum_i h_i^{(y)}(a_i) + \sum_{i<j} J_{ij}^{(y)}(a_i, a_j), \tag{S6}$$

and

$$Z_y = \sum_{\mathbf{a}} e^{H_y(\mathbf{a})}. \tag{S7}$$

It can be shown that Eqs. (S5) and (S6) follow as the least-biased form maximizing the entropy of genotype distribution under the constraints of given first and second moments, $f_i^{(y)}(a)$, $f_{ij}^{(y)}(a,b)$. By comparing Eqs. (1) and (2) in the main text, one may conclude that $-J_{ij}^{(y)}(a,b)$ must be the matrix inverse of the covariance $f_{ij}^{(y)}(a,b) - f_i^{(y)}(a)f_i^{(y)}(b)$. This is not exact because a_i is discrete, but in fact the mean field (MF) approximation (Sec. S1.7) leads to this conclusion. In general, the normalization constant Z_y, which corresponds to the determinant of covariance in normal distribution, causes difficulties in computation because of high-dimensional discrete summation necessary.

The set of parameters $\psi_y \equiv \{h_i^{(y)}(a), J_{ij}^{(y)}(a,b)\}$ for $a, b = 0, \cdots, l$ define the genotype distribution of the phenotype group y. They can be regarded as parameters that allow the genotype distribution to have the observed single-locus allele frequencies and correlation. Since the frequencies satisfy the relationships, $\sum_a f_i^{(y)}(a) = 1$, $\sum_a f_{ij}^{(y)}(a,b) = f_j^{(y)}(b)$, not all parameters among ψ_y are independent. Without loss of generality, one can set $h_i^{(y)}(0) = J_{ij}^{(y)}(0,b) = J_{ij}^{(y)}(a,0) = 0$ [68].

Strictly speaking, Eq. (S5) needs to be multiplied by a factor that accounts for the number of allelic combinations that can give rise to the given genotype $\mathbf{a}$: for instance, under the dominant model, $a_i = 1$ for a site i can arise from aA, Aa, and AA combinations. From this reasoning, one would write $\Pr(\mathbf{a}|y) = \prod_i g(a_i)\, e^{H_y(\mathbf{a})}/Z_y$, where $g(a) = 1, 3$ for $a = 0, 1$. However, it can be shown that this factor cancels out in the calculation of the main quantities of interest (likelihood ratio statistic and disease risk) considered below. We therefore omit this degeneracy factor in the derivation.

S1.3 Inference of genotype distributions

The actual inference of ψ_y is via maximum likelihood, for which we write the log likelihood term L_y of phenotype y in Eq. (S3) as

$$L_y = \sum_{k \in y} \ln \Pr(\mathbf{a}^k|y) - \frac{\lambda n_y}{2} \sum_{i<j} \sum_{a,b} J_{ij}^{(y)}(a,b)^2, \tag{S8}$$

where we added an l_2-penalizer acting on interaction terms for variable selection. Equation (S6) may be rewritten as

$$H_y(\mathbf{a}) = \sum_i \sum_a h_i^{(y)}(a)\delta(a_i, a) + \sum_{i<j} \sum_{a,b} J_{ij}^{(y)}(a,b)\delta(a_i,a)\delta(a_j,b), \tag{S9}$$

where $\delta(a,b) = 1$ if $a = b$ and zero otherwise, which gives

$$\frac{\partial H_y(\mathbf{a})}{\partial h_i^{(y)}(a)} = \delta(a_i, a), \quad \frac{\partial H_y(\mathbf{a})}{\partial J_{ij}^{(y)}(a,b)} = \delta(a_i,a)\delta(a_j,b). \tag{S10}$$

From Eqs. (S5), (S7), (S8), and (S10),

$$\frac{1}{n_y}\frac{\partial L_y}{\partial h_i^{(y)}(a)} = \hat{f}_i^{(y)}(a) - f_i^{(y)}(a), \tag{S11a}$$

$$\frac{1}{n_y}\frac{\partial L_y}{\partial J_{ij}^{(y)}(a,b)} = \hat{f}_{ij}^{(y)}(a) - f_{ij}^{(y)}(a,b) - \lambda J_{ij}^{(y)}(a,b), \tag{S11b}$$

where

$$\hat{f}_i^{(y)}(a) = \frac{1}{n_y}\sum_{k\in y}\delta(a_i^k, a), \tag{S12a}$$

$$\hat{f}_{ij}^{(y)}(a,b) = \frac{1}{n_y}\sum_{k\in y}\delta(a_i^k, a)\delta(a_j^k, b) \tag{S12b}$$

are the sample single-locus and pairwise frequencies and

$$f_i^{(y)}(a) = \frac{1}{Z_y}\sum_{\mathbf{a}}\delta(a_i, a)e^{H_y(\mathbf{a})}, \tag{S13a}$$

$$f_{ij}^{(y)}(a,b) = \frac{1}{Z_y}\sum_{\mathbf{a}}\delta(a_i, a)\delta(a_j, b)e^{H_y(\mathbf{a})} \tag{S13b}$$

are the corresponding population frequencies. For $\lambda = 0$, the maximum likelihood condition reduces to the requirement that the sample frequencies coincide with population frequencies.

Equations (S5), (S6), (S11), and (S12) can be extended to the pooled sample of all phenotypes by removing the restriction to phenotype group y. We denote these analogous quantities of the pooled group by symbols without the super(sub)script y. From Eq. (S3), the likelihood ratio test can be performed by first inferring distributions for all phenotype groups and then for the combined pooled group of n individuals with log likelihood

$$L_{\text{pooled}} = \sum_k \ln \Pr(\mathbf{a}^k) - \frac{\lambda n}{2}\sum_{i<j}\sum_{a,b} J_{ij}(a,b)^2 \tag{S14}$$

to obtain the statistic

$$q = 2\left(\sum_{y=0}^{Y} L_y - L_{\text{pooled}}\right). \tag{S15}$$

The degrees of freedom (d.f.) is given by the net change in the number of parameters; each ψ_y has $s = lm + l^2m(m-1)/2$ parameters, the two terms counting single-SNP and interaction parameters; d.f. $= (1+Y)s - s = Ys$.

To implement this in practice, one needs to evaluate the population frequencies given by Eqs. (S13) for a given set of parameters during the numerical maximization of L_y in Eqs. (S11). An obvious approach is the 'brute force' calculation, enumerating all genotypes to perform the summations, which is what we refer to as the exact enumeration (EE) approach. The number of terms in each summation is 2^m for dominant/recessive models, and EE is practical only for small m, but its results are exact in the limit of infinite sample size.

S1.4 Disease risk

The disease risk is obtained from genotype distributions via Bayes' theorem,

$$\Pr(y|\mathbf{a}) = \frac{\Pr(\mathbf{a}|y)p_y}{\Pr(\mathbf{a})} = \frac{\Pr(\mathbf{a}|y)p_y}{\sum_{y'}\Pr(\mathbf{a}|y')p_{y'}} = \frac{1}{1+\sum_{y'\neq y}\Pr(\mathbf{a}|y')p_{y'}/\Pr(\mathbf{a}|y)p_y}, \tag{S16}$$

which gives

$$\Pr(y|\mathbf{a}) = \frac{1}{1+e^{-F_y(\mathbf{a})}}, \tag{S17}$$

where

$$F_y(\mathbf{a}) = \ln \frac{\Pr(\mathbf{a}|y)p_y}{\sum_{y'\neq y}\Pr(\mathbf{a}|y')p_{y'}}. \tag{S18}$$

For the simple case-control scheme ($Y = 1$), Eq. (S18) reduces to the standard diseae risk expression: from Eqs. (S5), (S6), and (S18),

$$F_1(\mathbf{a}) = \alpha + \sum_i \beta_i(a_i) + \sum_{i<j}\gamma_{ij}(a_i, a_j), \tag{S19}$$

where

$$\begin{aligned}\alpha &= \ln \frac{p_1 Z_0}{p_0 Z_1}, && \text{(S20a)}\\ \beta_i(a) &= h_i^{(1)}(a) - h_i^{(0)}(a), && \text{(S20b)}\\ \gamma_{ij}(a,b) &= J_{ij}^{(1)}(a,b) - J_{ij}^{(0)}(a,b), && \text{(S20c)}\end{aligned}$$

and $a, b = 1, \cdots, l$. The parameter α controls disease prevalence $p_1 = 1 - p_0$, while $\theta \equiv \{\beta_i(a), \gamma_{ij}(a,b)\}$ represent single-locus and interaction effects to the disease risk, respectively. For the dominant/recessive model for which $l = 1$, each locus i has one parameter, $\beta_i \equiv \beta_i(1)$, and each site pair i, j also has one parameter, $\gamma_{ij} \equiv \gamma_{ij}(1,1)$, because the rest of parameters are zero by definition.

S1.5 Independent SNPs

If $J_{ij}^{(y)}(a,b) = 0$, the genotype distribution (S5) reduces to the categorical distribution with means $f_i^{(y)}(a)$:

$$\Pr(\mathbf{a}|y) = \prod_i \frac{e^{h_i^{(y)}(a)}}{z_{iy}}, \tag{S21}$$

where $z_{iy} = \sum_b e^{h_i^{(y)}(b)}$ and $Z_y = \prod_i z_{iy}$. Setting Eq. (S11a) to zero with $\lambda = 0$,

$$\hat{f}_i^{(y)}(a) = \frac{e^{h_i^{(y)}(a)}}{z_{iy}}. \tag{S22}$$

From $h_i^{(y)}(0) = 0$, $z_{iy} = 1/\hat{f}_i^{(y)}(0)$, and

$$h_i^{(y)}(a) = \ln \frac{\hat{f}_i^{(y)}(a)}{\hat{f}_i^{(y)}(0)}. \tag{S23}$$

Equations (S20) then become

$$\begin{aligned}\alpha &= \ln \frac{p_1}{p_0} + \sum_i \ln \frac{\hat{f}_i^{(1)}(0)}{\hat{f}_i^{(0)}(0)}, && \text{(S24a)}\\ \beta_i(a) &= \ln \frac{\hat{f}_i^{(1)}(a)/\hat{f}_i^{(1)}(0)}{\hat{f}_i^{(0)}(a)/\hat{f}_i^{(0)}(0)}, && \text{(S24b)}\end{aligned}$$

and $\gamma_{ij}(a,b) = 0$, $\hat{f}_i^{(y)}(0) = 1 - \sum_{a>0}\hat{f}_i^{(y)}(a)$. Equation (S24b) verifies the fact that for a single locus, the prospective and retrospective odds ratio are identical.

Equation (S8) reduces to

$$\begin{aligned}L_y &= \sum_{k\in y}\left[H_y(\mathbf{a}^k) - \ln Z_y\right] = \sum_{k\in y}\sum_i\left[\sum_a h_i^{(y)}(a)\delta(a_i^k, a) - \ln z_{iy}\right] = \sum_{k\in y}\sum_{i,a}\left[h_i^{(y)}(a) - \ln z_{iy}\right]\delta(a_i^k, a)\\ &= \sum_i\sum_a\sum_{k\in y}\delta(a_i^k, a)\ln \hat{f}_i^{(y)}(a) = n_y \sum_i\sum_a \hat{f}_i^{(y)}(a)\ln \hat{f}_i^{(y)}(a), \end{aligned} \tag{S25}$$

where Eq. (S9) was used, the identity $\sum_a \delta(a_i^k, a) = 1$ was inserted, and Eqs. (S22) and (S12a) were used. Analogously, we can write

$$L_{\text{pooled}} = n \sum_i \sum_a \hat{f}_i(a) \ln \hat{f}_i(a) = \sum_y n_y \sum_i \sum_a \hat{f}_i(a) \ln \hat{f}_i(a). \tag{S26}$$

Equation (S15) then becomes $q = \sum_i q_i$ with

$$q_i = 2 \sum_y n_y \sum_a \left[\hat{f}_i^{(y)}(a) \ln \hat{f}_i^{(y)}(a) - \hat{f}_i(a) \ln \hat{f}_i(a) \right]. \tag{S27}$$

The p-value p_i for site i is then

$$p_i = Q(l/2, q_i/2), \tag{S28}$$

where $Q(s,x)$ is the regularized gamma function. Equation (S27) has the form of Shannon entropy of mixing for Y groups.

The number of individuals with genotype a, $v_i^{(y)}(a) = \sum_{k \in y} \delta(a_i^k, a)$, follows multinomial distribution, and the population mean of a quantity can be evaluated as

$$\langle \cdots \rangle = \prod_{y=0}^{Y} \prod_{a=0}^{l} \sum_{v_y(a)=0}^{n_y} (\cdots) \binom{n_y}{v_y(0), \cdots, v_y(l)} \prod_{a=0}^{l} \hat{f}_i^{(y)}(a). \tag{S29}$$

For the dominant model, we used Eq. (S29) to calculate mean log odds ratio $\langle \beta_i(1) \rangle$ and power $\langle \Theta(q - q_c) \rangle$ where $\Theta(x) = 1$ for $x \geq 0$ and 0 otherwise (Additional file 1: Fig. S1).

S1.6 Pseudo-likelihood maximization

Pseudo-likelihood (PL) maximization gives better computational efficiency than EE. It is slightly less efficient than MF (Sec. S1.7) but more accurate. Along with EE, PL is also formally exact in the large n limit [71]. In our application, PL approximates Eq. (S8) using

$$P(\mathbf{a}^k | y) \simeq \prod_i P_i(a_i^k | y, \mathbf{a}_{j \neq i}^k), \tag{S30}$$

namely the product over all SNPs of the probability of genotype a_i^k at locus i conditional to the values of genotypes at other loci observed in the actual data set. This distribution is one-dimensional:

$$P_i(a | y, \mathbf{a}_{j \neq i}^k) = \frac{e^{\bar{h}_i^{(y)}(a)}}{\sum_b e^{\bar{h}_i^{(y)}(b)}}, \tag{S31}$$

where

$$\bar{h}_i^{(y)}(a) = \bar{h}_i^{(y)}(a; \mathbf{a}^k) = h_i^{(y)}(a) + \sum_{j \neq i} J_{ij}^{(y)}(a, a_j^k) \tag{S32}$$

is the effective field on site i given the genotypes of the observed data.

Equation (S8) becomes

$$L_y = \sum_i \sum_{k \in y} \left[\bar{h}_i^{(y)}(a_i^k) - \ln \left(\sum_b e^{\bar{h}_i^{(y)}(b)} \right) \right] - \frac{n_y \lambda}{2} \sum_{i<j} \sum_{a,b} J_{ij}^{(y)}(a,b)^2. \tag{S33}$$

The maximization of L_y can therefore be performed separately for each i. The first derivatives involved are

$$\frac{1}{n_y} \frac{\partial L_y}{\partial h_i^{(y)}(a)} = \hat{f}_i^{(y)}(a) - \frac{1}{n_y} \sum_{k \in y} P_i(a | y, \mathbf{a}_{j \neq i}^k), \tag{S34a}$$

$$\frac{1}{n_y} \frac{\partial L_y}{\partial J_{ij}^{(y)}(a,b)} = \hat{f}_{ij}^{(y)}(a,b) - \frac{1}{n_y} \sum_{k \in y} P_i(a | y, \mathbf{a}_{l \neq i}^k)\, \delta(a_j^k, b) - \lambda J_{ij}^{(y)}(a,b). \tag{S34b}$$

If $\lambda = 0$, Eqs. (S34) reduce to the condition that empirical frequencies coincide with the corresponding mean conditional probabilities averaged over the sample. For Z_y necessary for the calculation of α with Eq. (S20a), we found the following approximation to have best performance:

$$\ln Z_y = \frac{1}{n_y} \sum_i \sum_{k \in y} \ln \left[\sum_a e^{h_i^{(y)}(a) + \frac{1}{2} \sum_{j \neq i} J_{ij}^{(y)}(a, a_j^k)} \right]. \tag{S35}$$

In contrast to parameters derived from other methods, $J_{ij}^{(y)}(a,b) \neq J_{ji}^{(y)}(b,a)$ because of the asymmetry of Eq. (S34b) in PL. We took the mean values: $J_{ij}^{(y)}(a,b) \leftarrow [J_{ij}^{(y)}(a,b) + J_{ji}^{(y)}(b,a)]/2$.

S1.7 Mean field approximation

The main outcome of the MF approximation is summarized by the recipe for parameters ψ_y:

$$h_i^{(y)}(a) = \ln \frac{\hat{f}_i^{(y)}(a)}{\hat{f}_i^{(y)}(0)} - \sum_{j \neq i} \sum_b J_{ij}(a,b) \hat{f}_j^{(y)}(b), \tag{S36a}$$

$$J_{ij}^{(y)}(a,b) = -(\mathsf{C}_y^{-1})_{ij}(a,b) \quad \text{for } i \neq j,\ ab \neq 0, \tag{S36b}$$

where C_y is the $ml \times ml$ covariance matrix with elements $\hat{f}_{ij}^{(y)}(a,b) - \hat{f}_i^{(y)}(a)\hat{f}_j^{(y)}(b)$. By rearranging it, one may observe that Eq. (S36a) could be regarded as Eq. (S32) averaged over the genotype variables combined with Eq. (S23). In words, $h_i^{(y)}(a)$ in MF is the effective parameter acting on site i required to produce the observed allele frequencies at i and all other sites j. As described in Sec. S1.2, Eq. (S36b) is what one would expect if a_i were continuous normal random variables. We also need an approximation to $F_y \equiv \ln Z_y$:

$$F_y = -\sum_i \ln \hat{f}_i^{(y)}(0) - \sum_{i<j} \sum_{a,b} J_{ij}^{(y)}(a,b) \hat{f}_i^{(y)}(a) \hat{f}_j^{(y)}(b), \tag{S37}$$

which is used in calculating α with Eq. (S20a) as well as L_y.

Because the matrix C_y is often singular when m^2 is comparable to or larger than n, a regularization is needed. We replaced Eq. (S36b) with

$$J_{ij}^{(y)}(a,b) = -(\bar{\mathsf{C}}_y^{-1})_{ij}(a,b), \quad \bar{\mathsf{C}}_y = \epsilon\, \mathsf{C}_y + \frac{1-\epsilon}{ml} \mathrm{Tr}(\mathsf{C}_y) \mathsf{I}, \tag{S38}$$

where $\epsilon \in [0,1]$ and I is the identity matrix, such that $\mathrm{Tr}(\bar{\mathsf{C}}_y) = \mathrm{Tr}(\mathsf{C}_y)$. Note that $\mathrm{diag}(\mathsf{C}_y)_{ii}(a,a) = \hat{f}_i^{(y)}(a)[1 - \hat{f}_i^{(y)}(a)]$. As $\epsilon \to 0$, $\bar{\mathsf{C}}_y$ becomes diagonal and $J_{ij}^{(y)}(a,b)$ vanishes. Therefore, the independent-SNP limit is reached with $\epsilon = 0$ (no interaction), while $\epsilon = 1$ corresponds to the full interaction limit. To further help prevent singularities, for MF we modified Eqs. (S12) into

$$\hat{f}_i^{(y)}(a) = \frac{1}{1+n_y} \left[1/(l+1) + \sum_{k \in y} \delta(a_i^k, a) \right], \tag{S39a}$$

$$\hat{f}_{ij}^{(y)}(a,b) = \frac{1}{1+n_y} \left[1/(l+1)^2 + \sum_{k \in y} \delta(a_i^k, a)\delta(a_j^k, b) \right], \quad \text{for } i \neq j \tag{S39b}$$

[note that $\hat{f}_{ii}^{(y)}(a,b) = \delta(a,b)\hat{f}_i^{(y)}(a)$], which amounts to assuming a prior count of one individual with uniform genotype distribution.

In the rest of this subsection, we show the derivation of the formulas above, which is based on the expansion of Z_y for small coupling constants [68]. Equation (S7) is first generalized into

$$Z_y(\eta) = \sum_{\mathbf{a}} e^{H_y(\mathbf{a}, \eta)}, \tag{S40}$$

where

$$H_y(\mathbf{a},\eta) = \sum_i h_i^{(y)}(a_i) + \eta \sum_{i<j} J_{ij}^{(y)}(a_i,a_j), \tag{S41}$$

such that $\eta = 0$ yields independent SNP whereas $\eta = 1$ recovers the full model. The quantity $F_y = \ln Z_y$ is the moment-generating function for frequencies since, from Eqs. (S10) and (S13),

$$f_i^{(y)}(a) = \frac{\partial F_y}{\partial h_i^{(y)}(a)}, \tag{S42a}$$

$$f_{ij}^{(y)}(a,b) - f_i^{(y)}(a) f_j^{(y)}(b) = \frac{\partial^2 F_y}{\partial h_i^{(y)}(a) \partial h_j^{(y)}(b)} \equiv (\mathsf{C}_y)_{ij}(a,b). \tag{S42b}$$

One introduces a Legendre transform of F_y,

$$G_y = F_y - \sum_{i,a} h_i^{(y)}(a) f_i^{(y)}(a), \tag{S43}$$

a natural function of $f_i^{(y)}(a)$ rather than $h_i^{(y)}(a)$, for which

$$\frac{\partial G}{\partial f_i^{(y)}(a)} = -h_i^{(y)}(a), \tag{S44a}$$

$$\frac{\partial^2 G}{\partial f_i^{(y)}(a) \partial f_j^{(y)}(b)} = -(\mathsf{C}_y^{-1})_{ij}(a,b). \tag{S44b}$$

Equation (S44b) is obtained by differentiating Eq. (S44a) with respect to $f_j^{(y)}(b)$ and using Eqs. (S42).

From Eqs. (S23) and (S43), the zero'th order term of the expansion of $G_y(\eta)$ is

$$G_y(0) = F_y(0) - \sum_{i,a} h_i^{(y)}(a) f_i^{(y)}(a) = -\sum_{i,a} f_i^{(y)}(a) \ln f_i^{(y)}(a), \tag{S45}$$

whereas

$$\begin{aligned} \left.\frac{\partial G_y}{\partial \eta}\right|_{\eta=0} &= \left.\frac{\partial F_y}{\partial \eta}\right|_{\eta=0} - \sum_{i,a} \left.\frac{\partial h_i^{(y)}(a)}{\partial \eta}\right|_{\eta=0} f_i^{(y)}(a) \\ &= \frac{1}{Z_y} \sum_{\mathbf{a}} \left[\sum_{i<j} J_{ij}^{(y)}(a_i,a_j) + \sum_{i,a} \delta(a_i,a) \frac{\partial h_i^{(y)}(a)}{\partial \eta} \right] e^{H_y(\mathbf{a},0)} - \sum_{i,a} \frac{\partial h_i^{(y)}(a)}{\partial \eta} f_i^{(y)}(a) \qquad \text{(S46)} \\ &= \sum_{i<j} \sum_{a,b} J_{ij}^{(y)}(a,b) f_i^{(y)}(a) f_i^{(y)}(b). \qquad \text{(S47)} \end{aligned}$$

Therefore, to first order in η,

$$G_y(\eta) \simeq -\sum_{i,a} f_i^{(y)}(a) \ln f_i^{(y)}(a) + \eta \sum_{i<j} \sum_{a,b} J_{ij}^{(y)}(a,b) f_i^{(y)}(a) f_i^{(y)}(b). \tag{S48}$$

Applying Eq. (S44a) with $\eta = 1$, remembering that $\sum_a f_i^{(y)}(a) = 1$, and replacing $f_i^{(y)}(a)$ by its empirical estimate $\hat{f}_i^{(y)}(a)$, we get Eq. (S36a). From Eqs. (S44b) and (S48), Eq. (S36b) is obtained. From Eq. (S43), we get Eq. (S37).

S1.8 Logistic regression

If one instead introduces disease probability directly into Eq. (S1) via

$$\Pr(\mathbf{a}, y) = \Pr(y|\mathbf{a})p(\mathbf{a}), \tag{S49}$$

Equation (S1) becomes

$$L = \sum_{y}\sum_{k\in y} \ln \Pr(y|\mathbf{a}^k) + \sum_{k} \ln p(\mathbf{a}^k) \simeq \sum_{y}\sum_{k\in y} \ln \Pr(y|\mathbf{a}^k), \tag{S50}$$

where the marginal genotype distributions have been ignored in the last expression. For $Y = 1$, L is then a function only of θ if we postulate Eqs. (S17) and (S19), which corresponds to the standard logistic regression approach. Under this condition, Eq. (S50) can be written as

$$L(\theta) = \sum_{k} \left[y_k \ln \Pr(1|\mathbf{a}^k, \theta) + (1 - y_k) \ln P(0|\mathbf{a}^k, \theta)\right] - \frac{n\lambda}{2} \sum_{i<j}\sum_{a,b} \gamma_{ij}(a, b)^2, \tag{S51}$$

where we again added l_2-penalizer terms and $\Pr(1|\mathbf{a}^k, \theta)$ is given by Eqs. (S17), (S19), and (S20), and $\Pr(0|\mathbf{a}^k, \theta) = 1 - \Pr(1|\mathbf{a}^k, \theta)$. The maximization involves the derivatives

$$\frac{\partial L}{\partial \alpha} = \sum_{k} \left[y_k - \Pr(1|\mathbf{a}^k)\right], \tag{S52}$$

$$\frac{\partial L}{\partial \beta_i(a)} = \sum_{k} \delta(a_i^k, a) \left[y_k - \Pr(1|\mathbf{a}^k)\right], \tag{S53}$$

$$\frac{\partial L}{\partial \gamma_{ij}(a, b)} = \sum_{k} \delta(a_i^k, a)\delta(a_j^k, b) \left[y_k - \Pr(1|\mathbf{a}^k)\right] - n\lambda\gamma_{ij}(a, b). \tag{S54}$$

The likelihood ratio statistic of logistic regression is $q = 2[L(\theta) - A]$, since $A = \sum_y n_y \ln p_y$ is the log likelihood of Eq. (S51) under the null hypothesis.

S2 Supplementary Tables

Table S1: Indepdent-SNP inference comparison of logisitc regression (from PLINK) and DDA (GeDI).

Chr	SNP	Gene	Dominant model Odds ratio		Dominant model p-value		Genotypic model p-value	
			PLINK	DDA	PLINK	DDA	PLINK	DDA
1	rs7554267	*KCNT2*	0.6045	0.6045	8.152×10^{-12}	6.592×10^{-12}	6.534×10^{-12}	5.585×10^{-12}
1	rs10754196	*KCNT2*	0.4674	0.4674	1.401×10^{-24}	7.848×10^{-25}	1.212×10^{-25}	4.977×10^{-26}
1	rs800292	*CFH*	0.3433	0.3433	6.499×10^{-41}	2.850×10^{-41}	1.032×10^{-41}	1.855×10^{-43}
1	rs3766404	*CFH*	0.4032	0.4032	3.677×10^{-22}	4.303×10^{-22}	3.435×10^{-21}	3.670×10^{-21}
1	rs2019724	*CFH*	0.2518	0.2518	3.459×10^{-49}	2.224×10^{-57}	1.773×10^{-75}	3.643×10^{-85}
1	rs6677604	*CFH*	0.3669	0.3669	1.747×10^{-33}	1.380×10^{-33}	2.630×10^{-33}	6.548×10^{-34}
1	rs7517126		0.4141	0.4141	3.202×10^{-20}	3.925×10^{-20}	1.982×10^{-19}	1.860×10^{-19}
1	rs10801575	*CFHR4*	0.5388	0.5388	6.992×10^{-17}	5.639×10^{-17}	1.885×10^{-19}	1.179×10^{-19}
1	rs6667243	*CFHR5*	0.3206	0.3206	1.097×10^{-42}	1.527×10^{-46}	2.282×10^{-58}	2.390×10^{-63}
1	rs7555070	*CFHR5*	0.5217	0.5217	1.411×10^{-18}	8.693×10^{-19}	9.296×10^{-22}	4.403×10^{-22}
1	rs10754210	*F13B*	1.6030	1.6025	2.225×10^{-10}	2.272×10^{-10}	2.749×10^{-13}	4.529×10^{-14}
6	rs1042663	*C2*	0.3886	0.3886	8.280×10^{-18}	7.973×10^{-18}	6.264×10^{-17}	4.488×10^{-17}
6	rs550605	*C2*	0.3917	0.3917	1.263×10^{-17}	1.267×10^{-17}	9.324×10^{-17}	6.903×10^{-17}
6	rs429608	*SKIV2L*	0.4321	0.4321	1.828×10^{-21}	2.430×10^{-21}	1.126×10^{-20}	1.254×10^{-20}
10	rs6585827	*PLEKHA1*	0.5025	0.5025	2.308×10^{-16}	3.797×10^{-17}	6.278×10^{-21}	1.419×10^{-21}
10	rs7913140	*PLEKHA1*	0.7419	0.7419	1.876×10^{-4}	1.998×10^{-4}	6.544×10^{-4}	7.015×10^{-4}
10	rs2280141	*PLEKHA1*	0.5042	0.5042	2.302×10^{-16}	3.922×10^{-17}	7.049×10^{-21}	1.648×10^{-21}
10	rs2292627	*PLEKHA1*	1.9730	1.9730	3.359×10^{-16}	6.019×10^{-17}	3.656×10^{-16}	6.190×10^{-18}
10	rs2014307	*ARMS2*	0.5047	0.5047	1.696×10^{-19}	5.445×10^{-20}	5.910×10^{-21}	2.083×10^{-21}
10	rs2248799	*HTRA1*	0.4253	0.4253	2.669×10^{-24}	6.823×10^{-26}	1.538×10^{-28}	5.057×10^{-30}
