# Supplementary figures and images for: Genotype distribution-based inference of collective effects in genome-wide association studies: insights to age-related macular degeneration disease mechanism
Source: BMC Genomics. 2016 Aug 30;17(1):695. doi: 10.1186/s12864-016-2871-3 (PMC5006276; doi:10.1186/s12864-016-2871-3)

**a**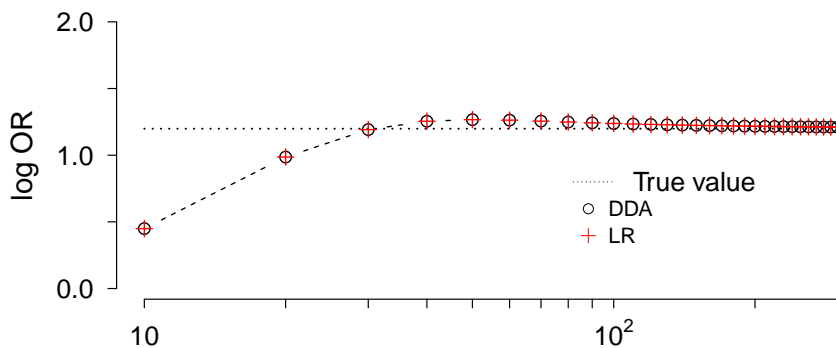**b**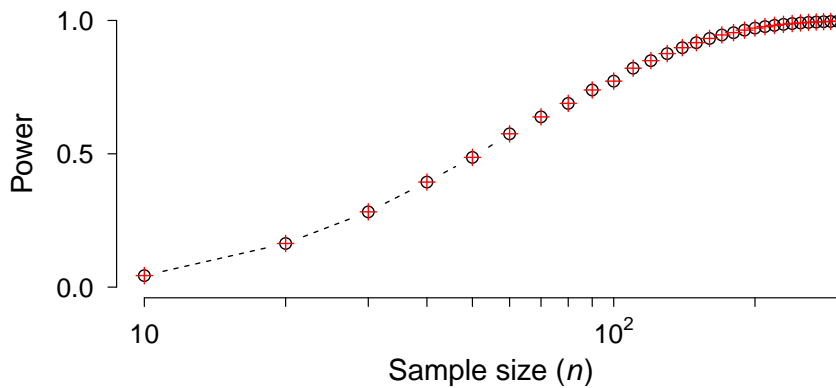

Supplement: Supplementary file 2 — Figure S1. Inference properties of a single independent SNP. Log odds ratio (OR) and power (level of significance 0.05) inferred from case-control data of size n=2n 0=2n 1 using DDA (analytic) and logistic regression (numerical) are shown for the dominant model. Equation (S29) of Text S1 was used. The minor allele frequency ϕ y for control and case groups were set such that $f^{(y)}=2\phi _{y}(1-\phi _{y})+{\phi _{y}^{2}}=\phi _{y}(2-\phi _{y})$f(y)=2ϕy(1−ϕy)+ϕy2=ϕy(2−ϕy). We used ϕ y=(0.1,0.25) such that f (y)=(0.19,0.4375) and h (y)=(−1.45,−0.25) for control and case groups, respectively, and β=1.1987. (PDF 6.99 kb) [file 12864_2016_2871_MOESM2_ESM.pdf]

**a**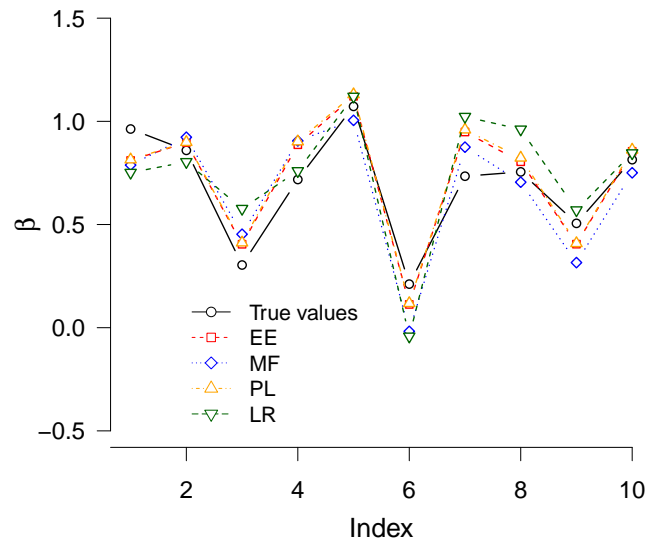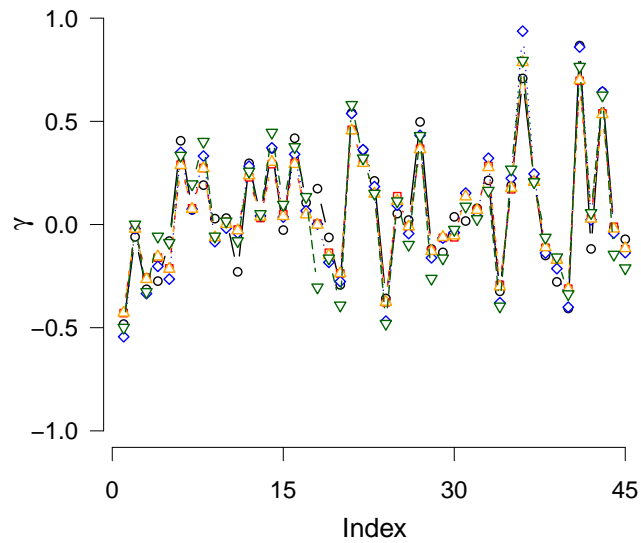**b**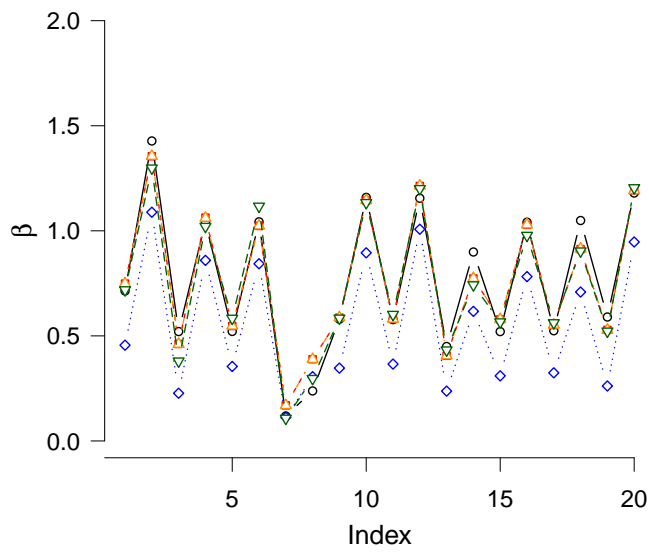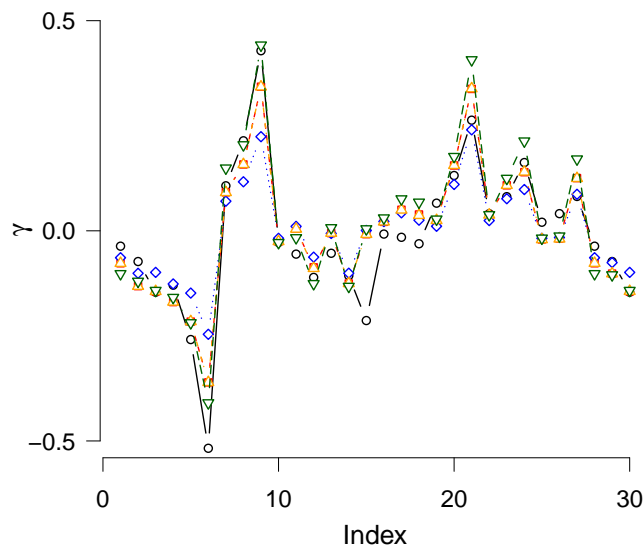

Supplement: Supplementary file 3 — Figure S2. Examples of true versus inferred parameters. a Dominant model with m=10 SNPs and inference on a sample of size n=103. b Genotypic model with m=10 SNPs and inference on a sample of size n=105. In all cases, the penalizer value was determined by cross-validation. (PDF 18.1 kb) [file 12864_2016_2871_MOESM3_ESM.pdf]

**a**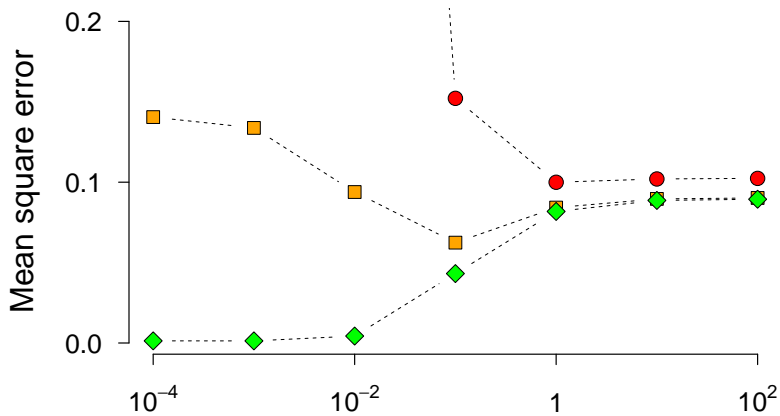**b**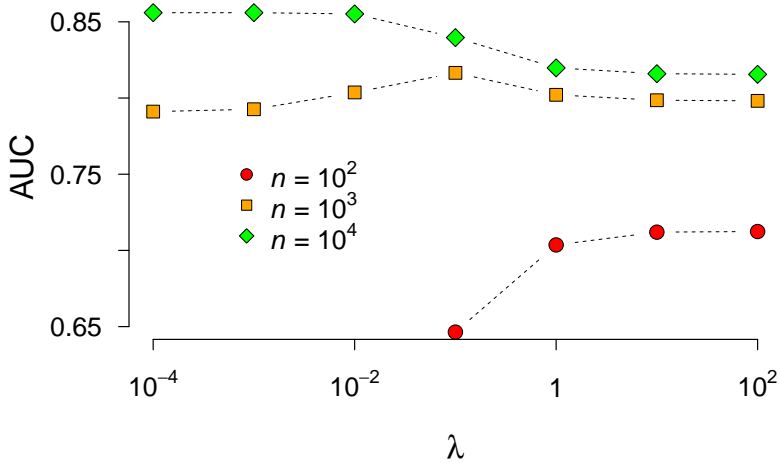

Supplement: Supplementary file 4 — Figure S3. Determination of penalizer λ via cross-validation. The data set is one realization of simulations shown in Fig. 2 b and the inference is with the exact enumeration (EE) method. The minima in mean square error (a) and the maxima in AUC (b) shift to lower λ as sample size n increases. (PDF 6.17 kb) [file 12864_2016_2871_MOESM4_ESM.pdf]

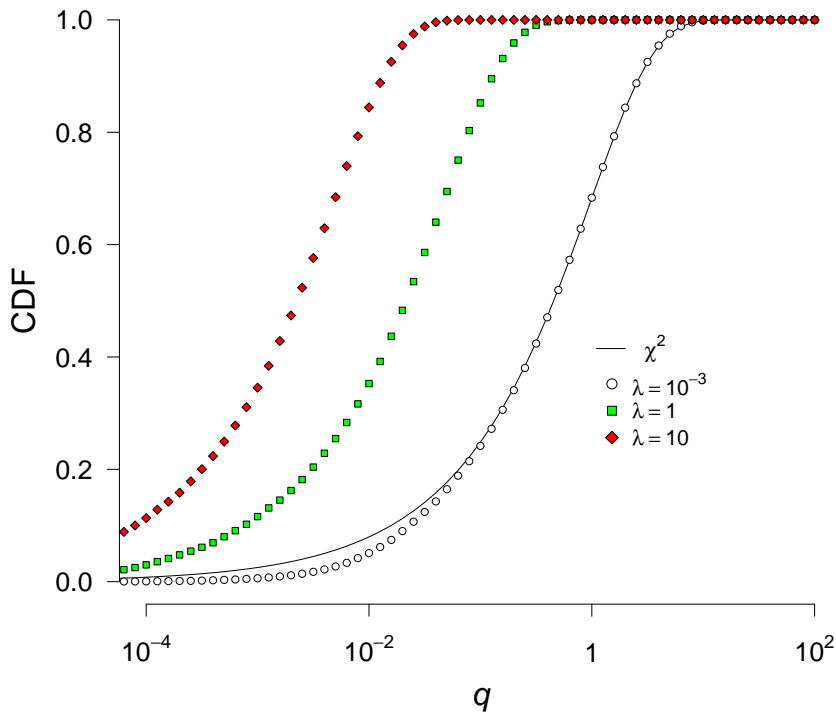

Supplement: Supplementary file 5 — Figure S4. Distributions of interaction likelihood ratio statistics under the null hypothesis. Empirical cumulative distribution functions (CDF) in terms of the interaction statistics q were obtained by resampling. Simulation conditions were as described in Fig. 2 e and inferences used EE. (PDF 7.39 kb) [file 12864_2016_2871_MOESM5_ESM.pdf]

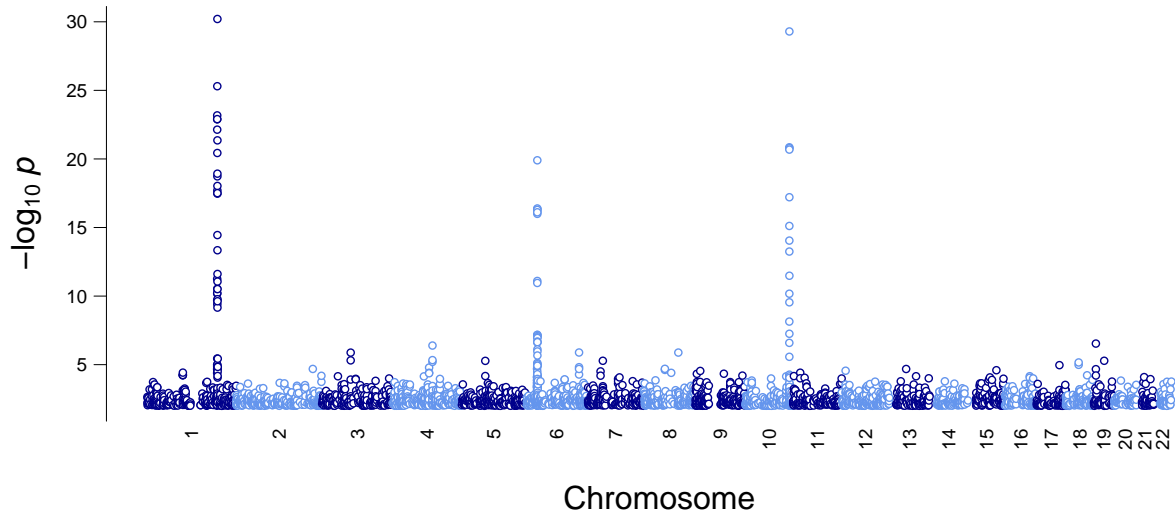

Supplement: Supplementary file 6 — Figure S5. Whole-genome p-value profile of AMD data. Independent-SNP DDA with genotypic model was used. (PDF 25.7 kb) [file 12864_2016_2871_MOESM6_ESM.pdf]

**a: CFH**

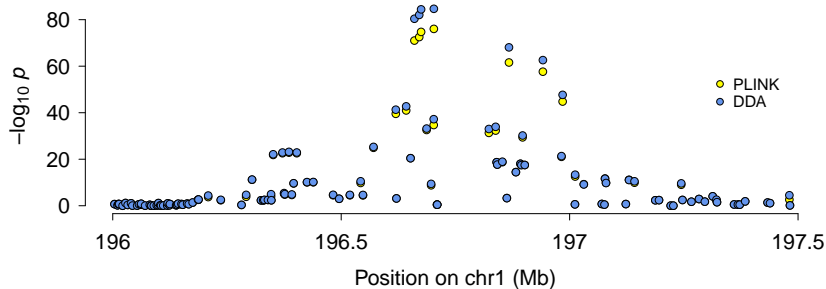

**B: C2/CFB**

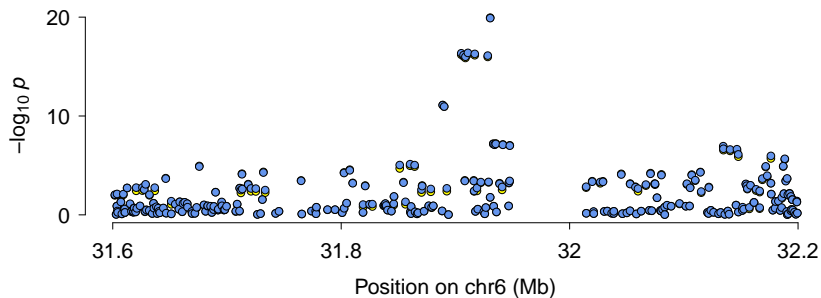

**B: ARMS2**

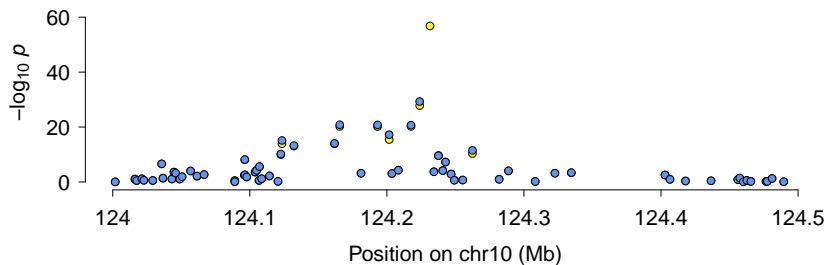

Supplement: Supplementary file 7 — Figure S6. Regional views of AMD data. Independent-SNP DDA results (light blue) are compared to logistic regression from PLINK (yellow). Genotypic model was used. (PDF 10.9 kb) [file 12864_2016_2871_MOESM7_ESM.pdf]

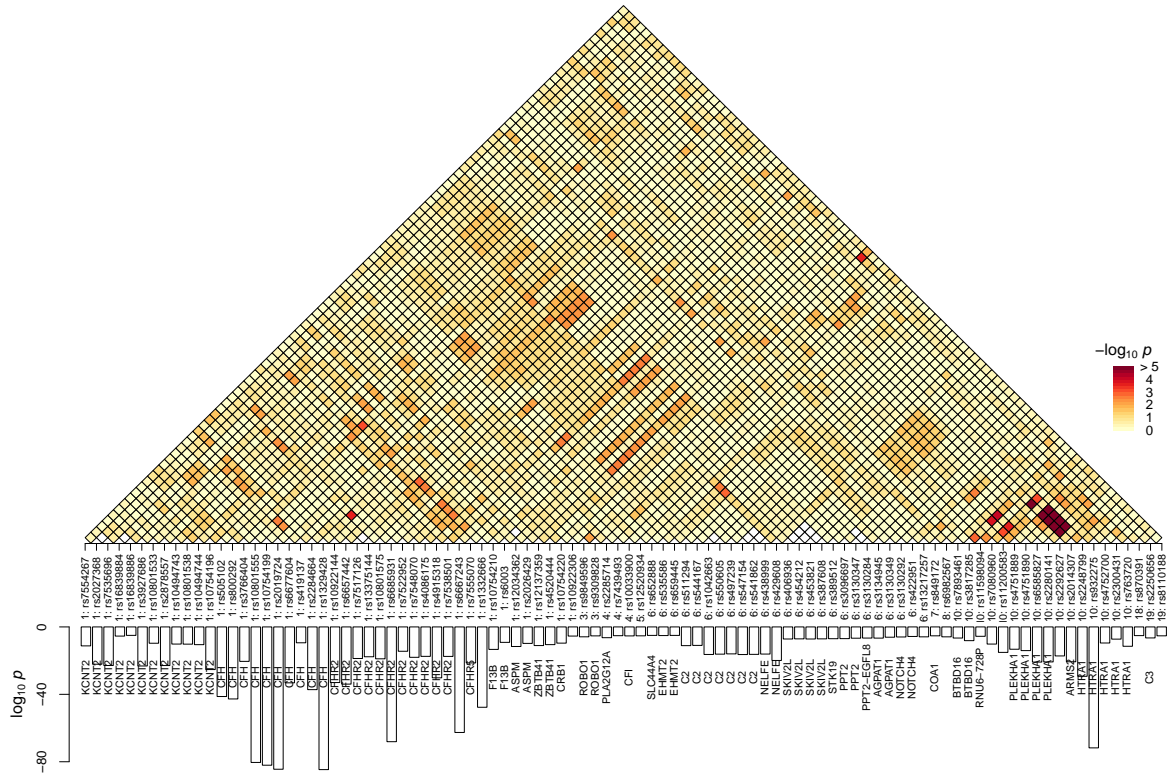

Supplement: Supplementary file 8 — Figure S7. Marginal pairwise interaction p-values. PLINK epistatic module was used to m=96 AMD SNPs. SNP pairs with strongest p-values near HTRA1 have p∼10−9. Genotypic model was used. The bottom panel shows the independent-SNP p-values. (PDF 54.6 kb) [file 12864_2016_2871_MOESM8_ESM.pdf]

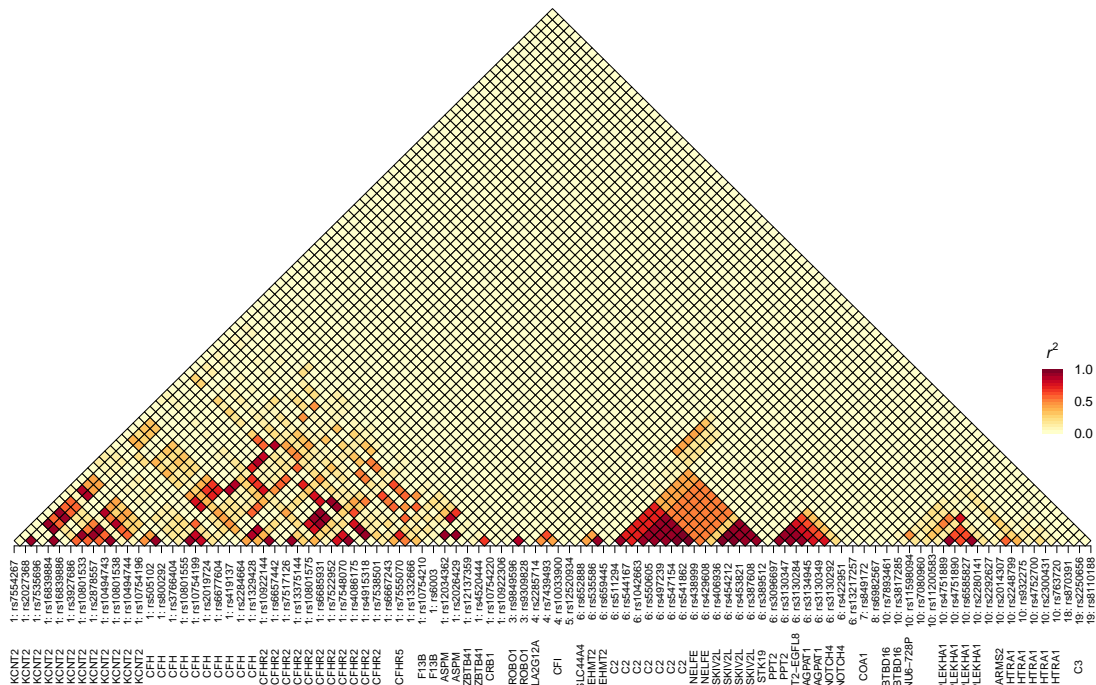

Supplement: Supplementary file 9 — Figure S8. Linkage disequilibrium r 2 within m=96 AMD SNPs from PLINK. (PDF 48.3 kb) [file 12864_2016_2871_MOESM9_ESM.pdf]
